# Knockdown of RNA interference pathway genes impacts the fitness of western corn rootworm

**DOI:** 10.1038/s41598-018-26129-6

**Published:** 2018-05-18

**Authors:** Courtney Davis-Vogel, Angel Ortiz, Lisa Procyk, Jonathan Robeson, Adane Kassa, Yiwei Wang, Emily Huang, Carl Walker, Amit Sethi, Mark E. Nelson, Dipali G. Sashital

**Affiliations:** 1 Research and Development, DuPont Pioneer, 7300 NW 62nd Ave., Johnston, IA USA; 2 Roy J. Carver Department of Biochemistry, Biophysics, and Molecular Biology, Iowa State University, 2437 Pammel Dr., Ames, IA USA

## Abstract

Western corn rootworm (*Diabrotica virgifera virgifera*) is a serious agricultural pest known for its high adaptability to various management strategies, giving rise to a continual need for new control options. Transgenic maize expressing insecticidal RNAs represents a novel mode of action for rootworm management that is dependent on the RNA interference (RNAi) pathways of the insect for efficacy. Preliminary evidence suggests that western corn rootworm could develop broad resistance to all insecticidal RNAs through changes in RNAi pathway genes; however, the likelihood of field-evolved resistance occurring through this mechanism remains unclear. In the current study, eight key genes involved in facilitating interference in the microRNA and small interfering RNA pathways were targeted for knockdown in order to evaluate impact on fitness of western corn rootworm. These genes include *drosha*, *dicer-1*, *dicer-2*, *pasha*, *loquacious*, *r2d2*, *argonaute 1*, and *argonaute 2*. Depletion of targeted transcripts in rootworm larvae led to changes in microRNA expression, decreased ability to pupate, reduced adult beetle emergence, and diminished reproductive capacity. The observed effects do not support evolution of resistance through changes in expression of these eight genes due to reduced insect fitness.

## Introduction

RNA interference (RNAi) is a biological process conserved across plants and animals wherein gene expression is controlled through a mechanism mediated by small 20–30 nucleotide complementary single-stranded RNAs^1^. The microRNA (miRNA) and small interfering RNA (siRNA) pathways provide endogenous control of gene expression, mobile genetic elements, and invading viruses^2–4^. Steps required for the interference response in various cellular contexts have been elucidated through study of model organisms, resulting in an advanced understanding of the RNAi pathways in the dipteran insect *Drosophila melanogaster*. The *D. melanogaster* miRNA pathway begins with the cleavage of endogenously expressed single-stranded primary miRNAs into precursor miRNAs by the microprocessor complex composed of Drosha and Pasha^5,6^. Precursor miRNAs are exported from the nucleus and further processed into short double-stranded RNA (dsRNA) duplexes by Dicer-1 (DCR-1) and Loquacious (LOQS)^7–9^. The *D. melanogaster* siRNA pathway is activated by long dsRNAs which can be taken up from the environment and processed into short duplexes in the cytoplasm by Dicer-2 (DCR-2) and R2D2^10,11^. One strand of either a miRNA or siRNA duplex is loaded into Argonaute 1 (AGO1) or Argonaute 2 (AGO2), respectively, forming active RNA induced silencing complexes (RISCs)^12–16^. Both types of RISC bind RNAs with some degree of complementarity to the guiding small RNA, resulting in repression or cleavage of target RNA^2,3^. These eight proteins—Drosha, Pasha, LOQS, DCR-1, DCR-2, R2D2, AGO1, and AGO2—are the core RNAi machinery of the mi- and siRNA pathways, so designated due to their central involvement in facilitating the interference response.

Both the mi- and siRNA pathways may be exploited to cause deliberate knockdown of essential genes in receptive organisms, a technique known as environmental RNAi (eRNAi) that has garnered interest in the agricultural sector as an innovative means of insect control^17^. Plant-produced RNAs have been demonstrated to provide protection against agricultural pests of several different phylogenetic orders^18–20^. The western corn rootworm (WCR – *Diabrotica virgifera virgifera*) is one of the costliest pests in North America, with an estimated $1.17 billion expended annually in management inputs and yield loss^21^. It was also the first major agricultural pest shown to be controllable through an RNAi-based transgenic trait^20^, and is the target of the first such trait registered by the United States Environmental Protection Agency^22^. Development of transgenic traits controlling WCR through novel modes of action such as RNAi are valuable due to the pest’s history of overcoming certain chemical and biological insecticides, as well as management practices such as crop rotation^23^. This adaptability must be taken into consideration prior to the deployment of new control technologies so as to maximize trait efficacy and lifespan through appropriate resistance management.

Development of WCR resistance to insecticidal RNAs has been speculated to be possible through upregulation of the target gene or target site insensitivity, though of particular concern would be the development of a resistance mechanism conferring protection across many or all insecticidal RNAs^23,24^. Such a mechanism would have to involve processes central to the RNAi response in insects. Artificially reducing expression of *dcr-2* and *ago2* has previously been reported to both confer complete protection to WCR adults against an insecticidal dsRNA and show no phenotypic effects in adults or larvae^25–27^. It was additionally shown that knockdown of *drosha* and *dcr-1* did not seem to affect WCR adults, though effects were observed with *ago1* knockdown^25–27^. Moreover, the siRNA pathway genes have been reported as among the 3% fastest evolving genes in *D. melanogaster*^28^, and eukaryotic organisms in general show high diversity in the presence, number, and function of RNAi pathway components^29^. Collectively, these reports imply that changes in certain core RNAi machinery may be sufficient to cause resistance to RNAi-based control in WCR. Recent evidence suggests evolution of RNAi pathway gene functionality in insects is a slow and complex process^30–33^. While certain regions of the sequences themselves may show rapid change, conserved regions that preserve protein function—and indeed the miRNA pathway genes themselves which participate in certain aspects of siRNA-mediated RNAi—show little to no evidence of positive selective pressure^28^. Despite hundreds of millions of years of host-pathogen interaction forcing adaptation in these sequences, including direct targeting by viral suppressors of RNA silencing, insects still rely heavily upon the RNAi pathway proteins for defense against entomopathogenic viruses^31,32,34,35^. Therefore, changes in expression of these genes was considered to be a more viable route to resistance than outright loss or functional mutation. The current study explores the effects of reduced expression of core RNAi pathway genes to determine whether this is a potential route to resistance.

Laboratory-based probes into broad theoretical resistance mechanisms can overlook potential repercussions of such mechanisms on practical viability of the insect. Many genes participating in the function and efficiency of insect RNAi pathways are involved in multiple processes, and their alteration may have widespread consequences—especially in a natural setting^35–37^. Downregulation of core components of the WCR RNAi pathway is potentially one of the most direct paths to resistance against RNAi-based control suggested to date. The current study provides an in-depth assessment of the impact on WCR of lowered core RNAi machinery expression. Knockdown targets include all eight genes serving core processing roles in the mi- and siRNA pathways, due to interest in utilization of both pathways for insect control, as well as their known or suspected pathway cross-functionality in *D. melanogaster*^7,38–44^. In agreement with previous reports, no phenotypic abnormalities were observed in larval stages upon treatment with dsRNA against these targets. However, knockdown occurring in older WCR larvae resulted in decreased ability to pupate, reduced adult emergence, and diminished reproductive capacity. Additionally, decreased expression of the core RNAi machinery caused changes in expression of miRNAs. The effects on post-larval WCR observed within this study argue against changes in expression of the core RNAi machinery directing field-evolved resistance.

## Results

### Design of WCR RNAi machinery knockdown experiments

**Figure 1:**
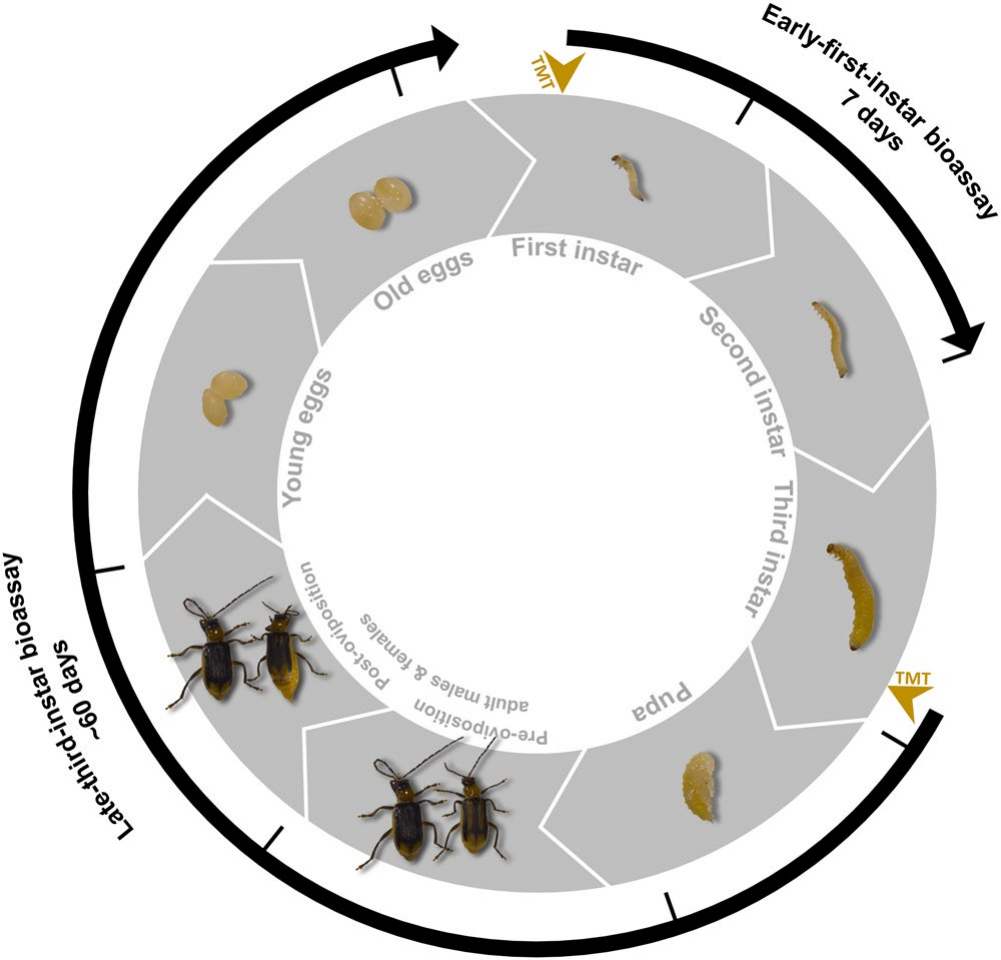
Bioassays used for knockdown of core RNAi machinery in relation to WCR life cycle. Depicted are eight points throughout the WCR life cycle relevant to the two bioassays used for exposure to dsRNA targeting core RNAi machinery: first through third larval instar, pupa, pre-reproductive adult male and female, reproductive adult male and female, young egg (<24 hours old), and old egg (<24 hours to hatch). In the early-first-instar bioassay, newly-hatched WCR larvae were placed on agar-based diet prepared with water for 24 hours and then transferred to fresh diet containing the treatments. Larvae were allowed to feed on the diet for seven days until late in the second larval instar, when they were assessed for growth and development. In the late-third-instar bioassay, larvae nearing the end of the third instar were placed on agar-based diet prepared with water for 24 hours, transferred to fresh diet containing the treatments for 24 hours, then placed into pupation medium. Treated larvae were monitored for transition to adults, survival through a designated reproduction period, capacity for egg production, and hatch rate of resulting eggs. Points at which insects were exposed to treatments (TMT) are indicated by gold arrows, and points at which they were collected for gene expression analysis are indicated by black lines. Diagram is not scaled to accurately represent insect size or time.

Double-stranded RNAs against each of the eight core RNAi machinery genes were prepared for oral administration to WCR larvae in two different types of bioassays (Fig. 1). To the extent possible, dsRNAs were designed to target all known isoforms of each gene^45^. The first type of bioassay utilized larval WCR early in the first instar. Approximately 24 hours post-hatch, larvae were presented with fresh diet containing either buffer, *Escherichia coli* β-glucuronidase (*gus*) dsRNA, *dvssj1* insecticidal dsRNA^46^, or target gene dsRNAs and allowed to feed for either two or seven days without diet refresh. At the end of each time point, larvae were collected for gene expression analysis when control larvae were late-first or late-second instar, respectively. At the end of the bioassay, larval growth and development was assessed prior to collection. Evaluation of the effects of each of the eight dsRNAs occurred over the course of several experiments, but may be directly compared.

The second type of bioassay utilized larval WCR late into the third instar. Approximately two to four days prior to pupation, actively feeding larvae were exposed for 24 hours to fresh diet containing each treatment. Treated larvae were then allowed to pupate and monitored for their ability to develop into reproductively capable adults. Insects were collected for gene expression analysis when control insects were late-third-instar larvae, pupae, adult males and females prior to and following a defined egg-laying period, and eggs approximately one day from hatch. Adult emergence, mortality, egg production, and egg hatch rate was measured. Due to assay complexity, exposure to the eight dsRNAs was split into two experiments. The first experiment was a pilot study with only a water and *dcr-1* dsRNA treatment, to ensure effects could be observed. The second experiment included additional negative controls and the remaining dsRNAs. Results should only be compared within an experiment due to variation in performance of the source WCR colony.

### Success and persistence of RNAi machinery knockdown

**Figure 2:**
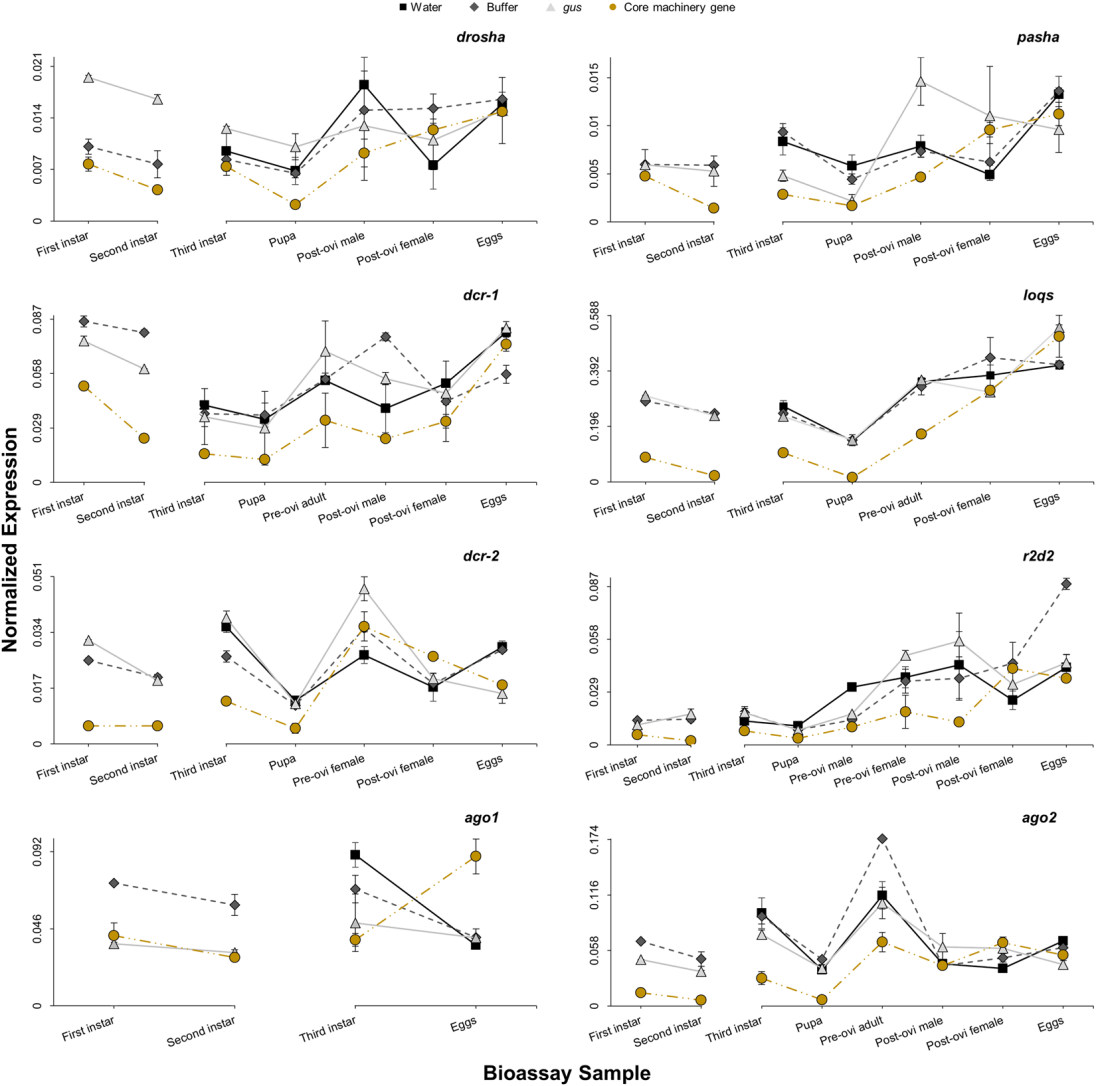
Knockdown of core RNAi machinery in early-first-instar and late-third-instar exposure bioassays. Insects were collected at 2 and 7 days post-treatment in the early-first-instar bioassays, when control larvae were first and second instar, respectively. Insects were collected at 3, 13, 30, 45, and 56 days post-treatment in the late-third-instar bioassays, when control insects were third instar, pupa, pre-oviposition adult male and female, post-oviposition adult male and female, and old egg, respectively. Samples (n = 3) were analyzed for expression of each core RNAi machinery gene with triplexed RT-qPCR using the reference genes *α-tubulin* and *ef1α*. Graphed points represent median normalized expression (±MAD), obtained from calculating the geometric mean of the ratios between interpolated concentration values of the target gene to each reference gene across all samples within each treatment group. All sample types were not available for all treatments at all collection points, depending on treatment effects. If expression for a particular target overlapped in the adult males and females across all treatments, results were combined and are indicated by the term “adult”. Expression in samples analyzed from the water treatment are shown in black squares (◼), from the buffer treatment in dark grey diamonds (), from the *gus* dsRNA treatment in light grey triangles (), and from the core machinery dsRNA treatment in gold circles ().

Effectiveness of the dsRNAs in suppressing each target gene was evaluated using reverse transcription polymerase chain reaction (RT-qPCR). Expression analysis was conducted on larvae collected from each treatment at both two and seven days post-exposure in early-first-instar bioassays, as well as at three days post-exposure in late-third-instar bioassays (Fig. 2). A decrease in transcript levels of each target gene is observed shortly after exposure for both first and third instar larvae, consistent with the robust response of WCR to eRNAi. Some targets show increased suppression after an additional five days in early-first-instar bioassays, while others show little difference in knockdown between two and seven days. Remaining samples collected from other points throughout the late-third-instar bioassays were also evaluated to monitor persistence of target knockdown (Fig. 2). Expression of most core RNAi machinery shows the greatest suppression in pupal samples (58–88% relative to buffer), then steadily recovers thereafter until reaching control treatment levels by the post-oviposition phase approximately 45 days following exposure. Eggs produced from insects treated with dsRNA directed against core machinery genes show no difference from control treatments, except for *ago1*-treated eggs which show increased expression of *ago1* transcript relative to controls.

### Knockdown causes minimal impact on WCR growth

**Figure 3:**
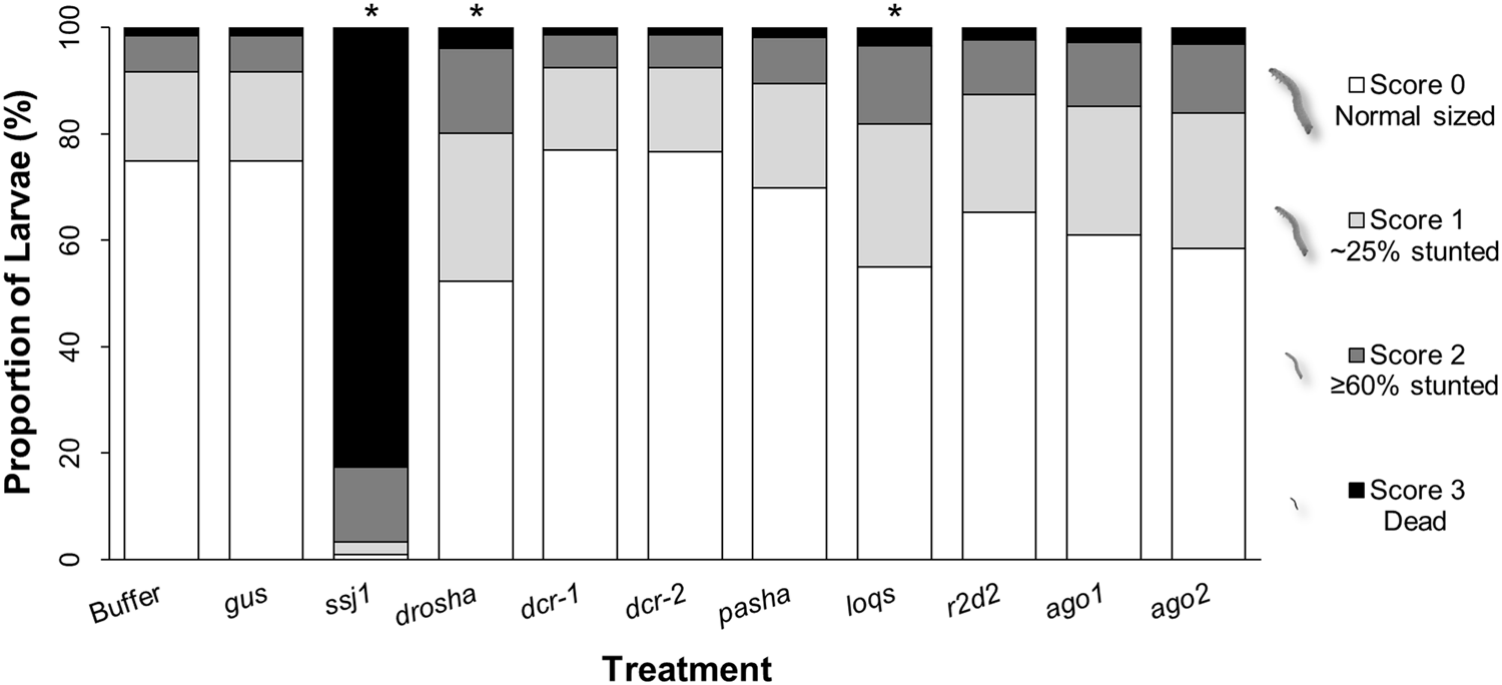
Growth of WCR larvae fed dsRNA targeting core RNAi machinery. All treatments were prepared on the start of each bioassay day using a target of 96 insects. Data represent percentage of insects in each treatment assigned to each category of impact, where 3 = mortality, 2 = severe stunting, 1 = stunting, 0 = no effect. Percent of insects scoring in each category was estimated from a generalized linear mixed model, and statistically significant differences were identified from Sidak-adjusted *P*-values. Treatments marked with an asterisk(*) indicate proportion of insects in each category is significantly different than the buffer and *gus* controls (*P* < 0.05). Results of development analysis of these insects are shown in Table 1.

Effect of the knockdown on growth (Fig. 3) and development (Table 1) of insects treated as early first instars was assessed at the end of a seven-day bioassay period (Fig. 1). The *dvssj1* positive control dsRNA shows strong effects during the bioassay period, causing over 90% mortality or significant developmental delay across all observations and in agreement with its previously characterized activity^46^. In contrast, most treatments containing dsRNA targeting core RNAi genes do not significantly differ from the negative controls despite substantial target knockdown. Only *drosha* and *loqs* show a mild but significant impact on growth but none on development during the seven-day bioassay period.

**Table 1:** Development of WCR larvae fed dsRNA targeting core RNAi machinery.

Treatment	Dead (%)	95% C.I (%)	*P*-value		First instar (%)	95% C.I (%)	Second & third instar (%)	*P*-value	
			(Buffer)	(*gus*)				(Buffer)	(*gus*)
Buffer	2.4	0.4–14.1	.	0.251	1.9	0.6–6.0	98	.	0.734
*gus*	6.2	1.1–28.1	0.238	.	3.7	1.5–8.6	96	0.707	.
dvssj1*	92	50.0–99.2	0.019	0.030	58.3	27.3–83.9	42	0.021	0.026
*drosha*	5.1	0.7–29.1	0.553	0.997	11.1	3.9–27.8	89	0.149	0.321
*dcr-1*	6.3	0.5–45.7	0.733	1.000	1.1	0.0–21.4	99	0.998	0.860
*dcr-2*	0.9	0.1–11.3	0.730	0.236	3.1	0.5–17.3	97	0.973	1.000
*pasha*	4.7	0.5–30.5	0.770	0.994	3.4	0.5–18.8	97	0.940	1.000
*loqs*	3.5	0.4–26.4	0.982	0.872	7.0	1.9–22.4	93	0.354	0.800
*r2d2*	7.9	1.1–40.7	0.290	0.994	2.5	0.3–19.8	98	1.000	0.998
*ago1*	6.9	1.0–35.1	0.275	1.000	3.6	0.6–19.6	96	0.919	1.000
*ago2*	1.6	0.2–14.6	0.982	0.315	6.7	1.8–21.5	93	0.377	0.841

All treatments were prepared on the start of each bioassay day using a target of 96 insects. Data represent larvae in each treatment showing mortality as percent of total infested larvae, and extent of development as percentage of live larvae. The combined proportion of second and third instar is composed of nearly all second instar larvae, as only five individuals across all treatments developed to third instar. Percent of insects within each developmental stage was estimated from a generalized linear mixed model, and statistically significant differences were identified from Dunnett-adjusted *P*-values. Treatments with *P*-values <0.05 are significantly different from negative controls and are marked with an asterisk(*). Results of growth analysis of these insects is shown in Fig. 3.

Late third instars treated with dsRNA against core RNAi machinery were collected and weighed in aggregated samples at select time points throughout the bioassay (Fig. 1). In general, no statistically significant differences are observed between treated and control insects (Fig. S1). Third instars treated with dsRNA against *drosha*, *loqs*, and *ago2* show decreased mass per insect, but only *loqs* is statistically different from negative controls. No significant differences are apparent in pupal, pre-oviposition female, or post-oviposition male masses. Pre-oviposition males were only measured for *dcr-1*-treated insects and no other core machinery gene due to a lack of spare males. The post-oviposition males that had been treated with dsRNA against *pasha* and females treated with *drosha*, *pasha*, and *ago1* dsRNAs show lower average masses, but the effect is not statistically significant. Knockdown of core RNAi machinery genes also does not cause a difference in mass of eggs produced, except those from *ago1*-knockdown insects which do show a significant increase.

### Decrease in larval gene expression reduces WCR pupation and emergence

**Figure 4:**
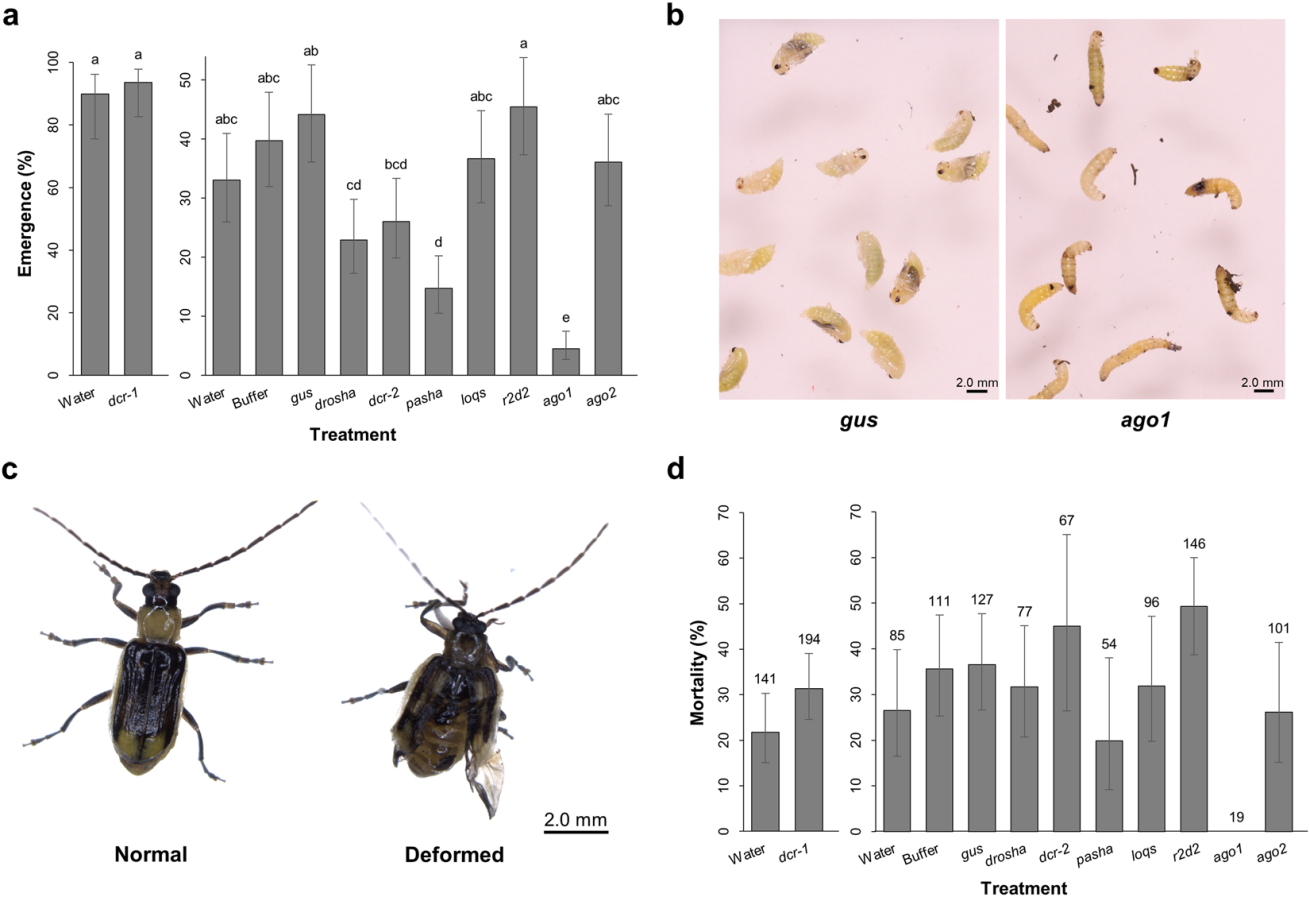
Effects of core RNAi machinery knockdown on WCR pupation and emergence. Data from both experiments of late-third-instar bioassay displayed in graphs represent means and 95% confidence intervals estimated using a generalized linear mixed model, and statistically significant differences identified from Tukey’s-adjusted *P*-values. Alphabetical letters indicating significance are shown for each treatment, and treatments followed by a common letter are not statistically different from each other at the significance level of 0.05. Results should only be compared within each experiment. (**a**) Emergence of treated WCR shown as a proportion of the total exposed larvae. Replication within treatment consisted of four pupation dishes each. (**b**) Phenotype displayed by *ago1*-treated insects. Images were collected 12 days following infestation of pupation dishes; most insects from this treatment were unable to pupate. (**c**) Phenotype displayed by some insects treated with *drosha*, *pasha*, or *ago1* dsRNAs—*ago1*-treated insects are shown as an example. These treatments showed higher incidence of wing and elytra malformations. (**d**) Adult mortality during the oviposition period. Replication within treatment consisted of three oviposition cages, with the exception of the *ago1* treatment (n = 1). As no significant differences on mortality across treatment were found, number of adult beetles is instead indicated above each bar.

Larvae treated as late third instars were allowed to pupate and adult beetles were counted and sexed as they emerged. In the second experiment, overall emergence and proportion of emerged males was lower than expected, but as this effect was observed across all treatments, it was attributed to performance of the WCR colony. The water negative control of the second experiment also showed reduced performance compared with the buffer and *gus* negative controls on certain parameters such as adult emergence. The unexpected discrepancy of this control does not compromise the overall conclusions, as the buffer and *gus* treatment results coincide and more accurately reflect conditions in the RNAi machinery knockdown treatments. Decreased expression of *drosha*, *dcr-2*, *pasha*, and *ago1* adversely affects the ability of WCR larvae to successfully transition to adults (Fig. 4a). The most severe reduction in emergence appears in *ago1*-treated insects, where most larvae are unable to pupate (Fig. 4b). Insects treated with dsRNA against *dcr-1*, *loqs*, *r2d2*, and *ago2* do not show differences in emergence rate compared to controls. The percentage of emerged males and females from treated larvae is not significantly different from control insects (Fig. S2), indicating that lowered expression of these genes had similar effects on both sexes.

### Emerged WCR adults show no increased mortality

Emerged WCR adults were next monitored for elevated mortality due to knockdown of core machinery genes. There were two monitoring periods: prior to female egg laying (pre-oviposition, through ~14 days post-emergence) and during female egg laying (oviposition, ~14 to 30 days post-emergence). Overall adult mortality was recorded during the post-emergence (pre-oviposition) period, while beetles dying during oviposition were sexed as they were recorded. No differences in post-emergence mortality are observed as a result of treatment (Fig. S2), although malformation of the elytra and wings are more prevalent in adults emerged from *drosha*- *pasha*- and *ago1*-treated larvae (Fig. 4c). Because no significant difference was detected in mortality of males versus females in any treatment, total adult mortality during the oviposition period was evaluated. No treatment significantly impacts adult mortality (Fig. 4d). Reduction in emergence of the *ago1*-treated insects was so extreme that only a few adults were available to assess post-emergence parameters, making the absence of mortality in these insects biologically meaningless.

### Larval gene knockdown inhibits WCR fecundity

Ability of the emerged WCR adults to produce viable eggs was examined for effects resulting from reduced core machinery expression. Following the pre-oviposition emergence period, oviposition cages were established by treatment and calibrated to the number of available females. Differences in emergence rates affected the ability to reach target rates of females and males per cage for some treatments. The presence of fewer males than females per cage is not expected to influence the measure of overall reproductive capacity. Adults mated during both the pre-oviposition and oviposition periods, so eggs produced within a cage do not only reflect matings between the females and males in that specific cage. The oviposition period was broken into three time points, and eggs were collected from each cage at the end of each time point, pooled by treatment, incubated, aliquoted, and finally monitored for hatch.

**Figure 5:**
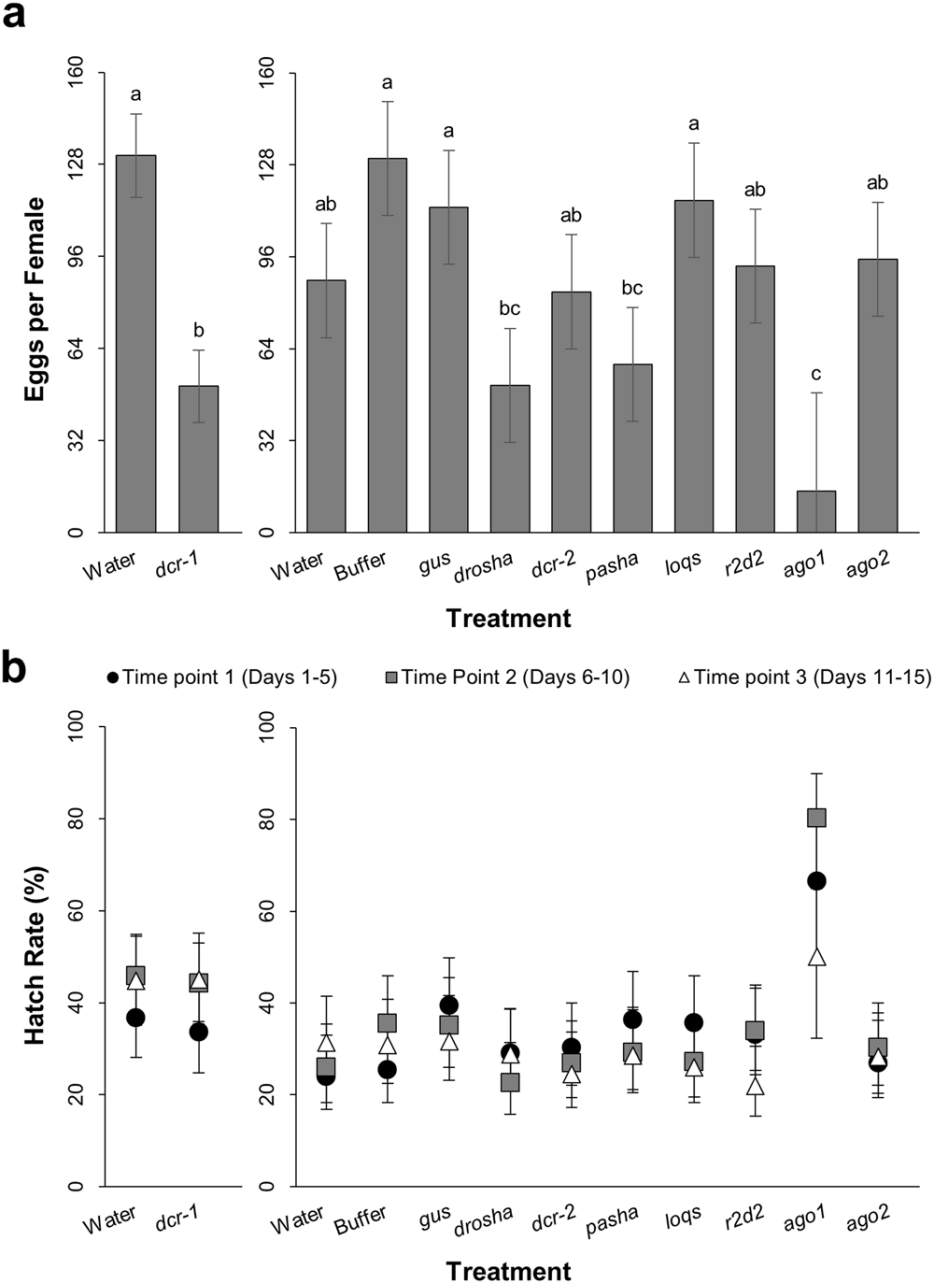
Effects of core RNAi machinery knockdown on WCR egg production and egg hatch rate. Data from both experiments of late-third-instar bioassay displayed in graphs represent means and 95% confidence intervals estimated using a linear mixed model (egg production) or generalized linear mixed model (hatch rate), and statistically significant differences identified from Tukey’s-adjusted *P*-values. Alphabetical letters indicating significance are available in Supplementary File 1 for each treatment, where treatments followed by a common letter are not statistically different from each other at the significance level of 0.05. Results should only be compared within each experiment. (**a**) The number of eggs produced during the first oviposition time point per starting number of WCR females. Replication within treatment consisted of three oviposition cages, with the exception of the *ago1* treatment (n = 1). (**b**) Egg hatch rate across time points 1 (black circles ●), 2 (grey squares ), and 3 (white triangles Δ) shown as percent hatch of total tested eggs. Replication within treatment consisted of three aliquots of eggs per cage per time point (n = 9 per time point, *ago1* n = 3 per time point). Significant difference from the buffer control was observed in eggs whose parents had been treated with *ago1* dsRNA at time points 1 and 2, and from the *gus* control in *ago1* eggs at time point 2 (*P* < 0.05).

Calculation of egg production per starting oviposition female during the first time point reveals that treatment with dsRNA against *dcr-1*, *drosha*, *pasha*, and *ago1* causes drastic reductions in numbers of eggs laid (Fig. 5a). A lower capacity for egg production is also apparent in females treated with *dcr-2*, *r2d2*, and *ago2* dsRNAs, though these treatments are not statistically different from the buffer and *gus* controls. No effect is observed for the number of eggs produced by females treated as larvae with *loqs* dsRNA. A similar pattern of treatment effects is replicated within the other oviposition time points and regardless of the calculation method used (Fig. S3a–d). Egg hatch exhibited a time-dependent effect, so percent hatch was calculated separately for each treatment across all three oviposition time points (Fig. 5b). No treatment causes statistically significant effects on hatch rate except *ago1*, where eggs show a 20–30% increased hatch rate relative to the buffer and *gus* controls in all three time points. Together, these results indicate the primary contributor to the observed reduction in WCR fecundity is reduced egg production, with minimal to no contribution from decreased egg viability.

### Knockdown impedes proper expression of WCR miRNAs

**Figure 6:**
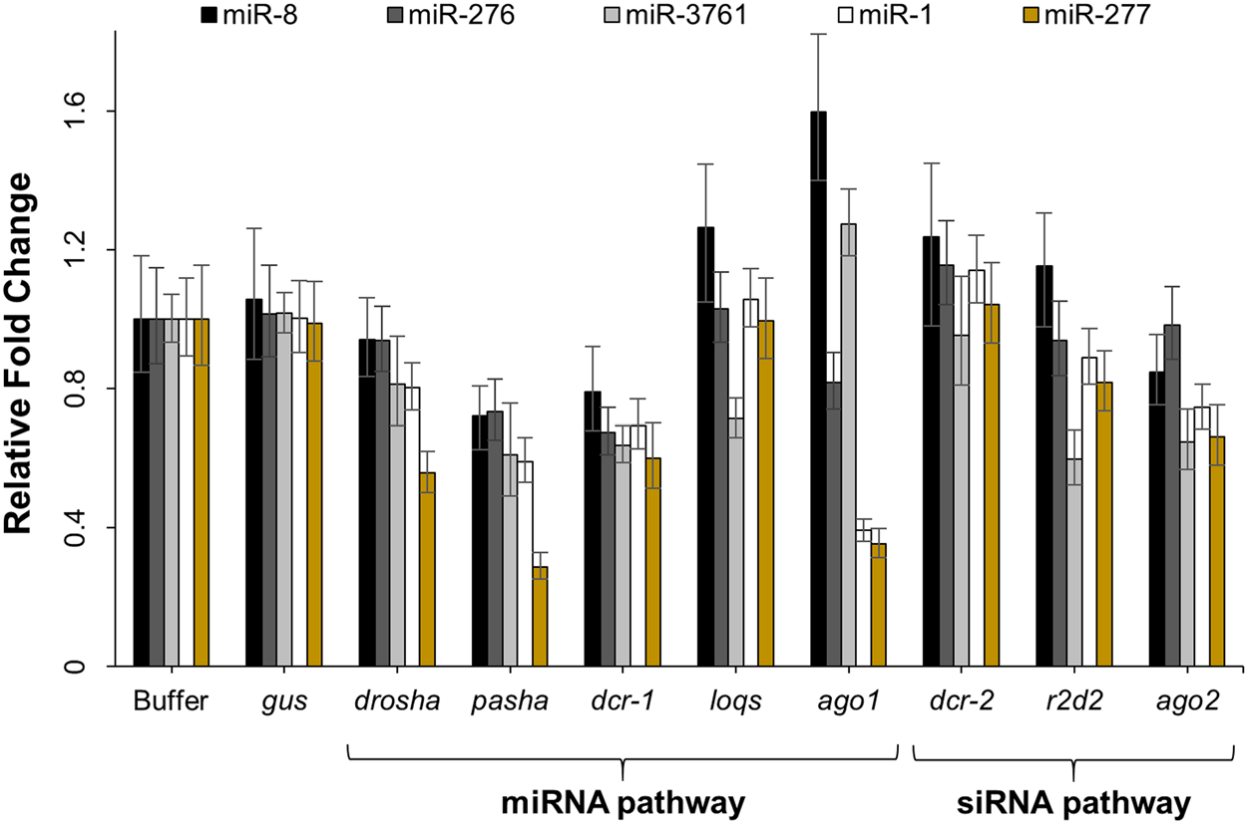
Effects of core RNAi machinery knockdown on miRNA expression in WCR larvae. Insects collected 7 days post-treatment in the early-first-instar bioassays were analyzed for expression of three probable AGO1-loaded miRNAs (miR-8–3p, miR-276, miR-3761) and two probable AGO2-loaded miRNAs (miR-1a-5p, miR-277). Samples were those described in Fig. 2 (n = 3), and were analyzed for expression of each miRNA with two-step singleplex RT-qPCR. Graphed bars represent mean relative fold change (±SD), obtained using the 2^(−ΔΔCt)^ method with a spiked RNA reference and buffer-treated samples as the control group. From left to right for each treatment, expression of miR-8 is shown in black, miR-276 in dark grey, miR-3761 in light grey, miR-1 in white, and miR-277 in gold.

Because phenotypic effects were not obvious at certain WCR stages despite confirmed knockdown of core RNAi machinery genes, functionality of the RNAi pathways was assessed by examining expression levels of five diagnostic miRNAs. These miRNAs were chosen based on reports in *D. melanogaster* that they are preferentially loaded into AGO1 (miR-8, miR-276, likely miR-3761) or AGO2 (miR-1 and miR-277)^43^, or were found to consistently express across the WCR life cycle (all five – unpublished data). MicroRNA expression was evaluated by RT-qPCR. Knockdown of *drosha*, *pasha*, *dcr-1*, *loqs*, *ago1*, *r2d2*, and *ago2* changes expression of one or more miRNAs compared with control insects (Fig. 6). These changes were observed in second instar larvae collected seven days after dsRNA treatment in the early-first-instar exposure bioassays. However, little to no effect on expression of these miRNAs is observed in pupae collected from the late-third-instar exposure bioassays (Fig. S4).

## Discussion

Next-generation insect control based on RNAi technology will be an important component in the continued arms race with agricultural insect pests. In order to protect this new technology, it is beneficial to understand potential paths through which insects may develop resistance and proactively incorporate management strategies into their deployment. High levels of resistance to insecticidal proteins are usually receptor-mediated and therefore specific to a single protein or a small number of proteins which bind the same receptor^37^. Insecticidal RNAs—regardless of target—depend on a highly conserved central processing mechanism within the pest for efficacy. A scenario can be imagined where insects may develop resistance to all insecticidal RNAs through changes in expression or function of proteins involved in RNAi pathways. However, ambiguity exists about whether changes in RNAi machinery expression would result in fitness costs that would limit establishment of a resistant population. Western corn rootworm is the primary target of transgenic maize expressing insecticidal RNAs^46,47^, and has well-documented adaptability to many pest management tactics^48–50^. The current study reveals fitness costs associated with reduced expression of the WCR core RNAi machinery, suggesting this may be an unfavorable path to eRNAi resistance.

Two types of bioassays were used to mimic a possible resistance paradigm where expression of core RNAi machinery is reduced in larvae, thereby theoretically conferring protection against insecticidal RNAs to vulnerable life stages. In agreement with and expansion of a previous report^25^, the first type of bioassay confirmed exposure of WCR first instar larvae to dsRNA targeting *dcr-2* and *ago2* resulted in no overt phenotypic changes despite robust gene suppression. Knockdown of an additional six core mi- and siRNA pathway genes also showed little or no phenotypic impact. However, effects on the expression of several miRNAs were noted in all treatments except *dcr-2*. Most of these proteins are known to participate early in *D. melanogaster* development, and have maternal contributions of either gene or protein products^51–57^. Additionally, these transcripts exhibit periods of increased expression during aging of *D. melanogaster* eggs^58,59^. A recent report showed similar increased expression in WCR eggs^45^. High endogenous expression during egg development and long-lived mRNA or proteins may explain why knockdown of these genes lack overt larval phenotypes. Carryover of embryonic RNAi machinery mRNA or protein may also disallow protection against insecticidal RNAs in a field setting, as it may be difficult to decrease expression of these critical developmental genes in time to provide protection.

In a second type of bioassay, consequences of core RNAi machinery knockdown in larvae were seen to manifest later in the WCR life cycle. Reducing expression of all eight core machinery genes in late-third-instar larvae led to a variety of phenotypes that could negatively impact field viability, most strikingly adult emergence and egg production. Decreased expression of *drosha*, *dcr-2*, *pasha*, and *ago1* in WCR larvae preparing to pupate reduced their ability to successfully transition to adults. Expression of these four transcripts increases from third instar to pupa^45^, and dsRNA treatment just prior to this phase in many cases seems to reduce protein abundance below a level necessary for completion of metamorphosis. A similar expression pattern was also reported for *dcr-1* and *ago2*, but knockdown of these two targets did not significantly influence adult emergence. Expression of *r2d2* was not reported to change between third instar and pupa, and likewise did not noticeably disrupt emergence. These proteins may not be as critical for transition through the pupal stage; they may also turn over more slowly than the others, making protein already present prior to dsRNA suppression sufficient for the transition, especially if they are most necessary in pre- or early pupation. Larvae treated at third instar successfully emerging as adults additionally showed diminished egg-laying capacity. Targeting *drosha*, *dcr-1*, *dcr-2*, *pasha*, *ago1*—and to a lesser but obvious extent *r2d2* and *ago2*—caused WCR females to produce lowered numbers of eggs. All of these genes contribute to normal fertility in *D. melanogaster* either through formation of ovary architecture or during gametogenesis^51,60–62^. If they serve analogous functions in WCR reproduction, inadequate protein to fulfill these roles could explain the observed reduction in numbers of eggs produced by WCR females.

Considering the presumed involvement of the WCR core RNAi pathway machinery in developmental processes, it is not surprising that effects were observed upon suppression of most of these targets. However, effects of *loqs* knockdown are conspicuously absent. Treatment with *loqs* dsRNA in WCR only slightly reduces the size of second and third instar larvae. If this protein is involved in processing exogenous dsRNAs in WCR, reducing its expression may represent a possible path to eRNAi resistance for WCR. Reports in other insects suggest such a functionality in processing exogenous dsRNAs for LOQS^39,63^, though its role in the siRNA pathway is currently thought to predominate in the processing of endogenous siRNAs^64,65^. Characterization of LOQS as a core component of the *D. melanogaster* miRNA pathway has also been recently questioned; it has been reported as important late in pupation and ovary and ovariole formation, but some individuals survive the absence of this transcript and not all miRNAs require it for maturation^53,62,66–68^. The relevance of *loqs* expression to the WCR RNAi pathways and eRNAi requires further study.

It is important to note the timing and manner of dsRNA exposure may have affected the effects observed in the current study. Again, longevity of RNAi machinery mRNAs and proteins in WCR is unknown and may be masking the true consequences of reduced gene expression. Because knockdown was instigated by only one dsRNA exposure and was not refreshed, suppression did not persist at full strength, evidenced by expression recovery to normal levels in post-oviposition adults and eggs. This may also explain why essentially no effects were seen in eggs, despite potential maternal contributions to egg development, as well as previously reported successful parental RNAi in rootworm^69^. Comparing the results reported here with a recent study examining effects of repeated exposure of dsRNAs targeting five core RNAi genes on WCR adults illustrates the importance of timing in these bioassays^27^. Most targets examined did not show any effects in that study. However, similar to the current work, it was found that *ago1* knockdown decreased adult survival and egg production. Interestingly, although the previous study found that egg hatch was also decreased upon *ago1* knockdown, in the current study the small number of eggs laid by females emerged from *ago1*-treated larvae were larger, hatched at elevated rates, and were the only observed instance of transgenerational expression change. A bioassay design where knockdown was consistently maintained from egg hatch through adult oviposition would likely reconcile the differences between the two studies. New or enhanced effects of reduced expression of core RNAi machinery may be revealed under such conditions, including additional transgenerational effects.

This and previously reported studies have explored potential WCR resistance to RNAi-based traits through lowered core RNAi machinery expression under laboratory conditions. It has been shown that fitness costs detected in a laboratory setting are exacerbated in the field due, among other factors, to the presence of plant phytochemicals and entomopathogens^37,70,71^. The critical role of the siRNA core machinery in immune responses against not only viruses, but bacteria and fungi, has been demonstrated in *D. melanogaster* and other insects^35,72,73^. If these genes function similarly in WCR, reduced expression of core RNAi components in young larvae may increase susceptibility to soil-based entomopathogens and other stressors. In such a case, effects that are minimized or silent in the laboratory, like those observed here regarding miRNA expression changes in larvae, may become deleterious in the field. It could also be possible that suppression specifically in WCR gut epithelium may provide protection against insecticidal dsRNAs while leaving the majority of cells with unchanged levels of these essential proteins. Arguably negating this possibility is the fact that oral exposure is a primary route of insect infection by entomopathogens, specifically viruses^74^. It has also been shown in at least one insect that a gut-specific upregulation of siRNA pathway genes occurs in response to persistent viral infection^75^. Little has been reported concerning pathogens infecting WCR, though several rootworm viruses have recently been characterized^76–78^. Further research regarding the nature of WCR entomopathogens, mode of infection, and interaction with RNAi machinery is needed to clarify this point.

Changes in key RNAi processing components has been suggested as a route through which WCR may develop resistance to eRNAi^24,25,27^. This study has shown that lowered expression of core mi- and siRNA genes in WCR larvae perturb miRNAs and lead to reductions in mass, adult emergence, and fecundity—factors that could disfavor survival of these insects in the field. Due to observed impacts on fitness and the requirement of their *D. melanogaster* homologues for various developmental processes and vigor in a natural environment, significant changes in the WCR core RNAi pathway genes seem unlikely to independently enable field-evolved resistance. Though the current study cannot account for co-evolution of compensatory mechanisms that promote tolerance to lowered expression of these genes, changes in alternative gene candidates having no significant involvement in development and viral defense may be simpler to achieve and therefore more advantageously facilitate development of eRNAi resistance. Studies in other insects indicate that genes involved in the uptake or degradation of exogenous RNAs may be such candidates^79–84^. Because a population showing field-evolved resistance to this new technology is not currently available, research on other proposed mechanisms should also consider impact on insect fitness, preferably throughout the entire insect life cycle and in a setting that feasibly mimics field conditions. This research is critical for incorporating appropriate strategies for mitigation of resistance into the deployment of this important new insect control technology.

## Methods

### Production of dsRNA

Double-stranded RNAs were produced by first designing a set of primers for each target containing the T7 RNA polymerase start sequence appended to the 5’ end of each external primer. Target-specific primers were used to amplify from complementary DNA (cDNA) a 500 bp fragment of *dcr-1* and *dcr-2*. Employing standard methods^85^, overlapping primers were used to self-amplify a synthetic 277 bp fragment of the following targets: *E. coli gus* (GenBank accession number S69414.1), *drosha*, *pasha*, *loqs*, *r2d2*, *ago1*, and *ago2*. Template PCR was conducted using Invitrogen Platinum PCR SuperMix High Fidelity; primers and PCR conditions are outlined in Table S1. Template produced from cDNA was separated on 1.2% agarose gels and purified using the QIAquick Gel Extraction Kit with associated manufacturer’s instructions. Template produced from overlapping primers was used without purification. Large-scale (100-reaction) *in vitro* transcription (IVT) was carried out for each target using the Ambion MEGAscript T7 Transcription Kit and 400 µL template following manufacturer’s protocol with an overnight 37 °C incubation. Following DNase treatment, a subsample of each reaction was purified using the Ambion MEGAclear Transcription Clean-Up Kit, confirmed by gel electrophoresis as correctly sized, and quantified using spectroscopy. Resulting subsample concentrations were used to back-calculate concentrations of the large-scale IVT reactions.

### Early-first-instar exposure bioassays

Early-first-instar bioassays were conducted in five experiments, each containing three to six treatments: two negative controls (buffer & *E. coli gus* dsRNA), a positive control (*dvssj1* dsRNA), and one of each dsRNA targeting expression of a core RNAi machinery gene. Overall diet preparation method followed a modified version of the artificial diet manufacturer’s *Diabrotica* recommendations (Frontier Scientific, Inc., Newark, DE), and insects were reared and handled by an internal insectary as previously described^86,87^. Two 96-well diet plates per treatment per experiment were prepared as previously described^46^, with buffer-normalized (v·v^−1^) dsRNA dosing solutions containing 300 ng·µL^−1^ resulting in final diet concentrations of 50 ng·µL^−1^. Newly-hatched WCR were acclimatized to neutral diet for 24 hours prior to transfer to treatment diets at a target rate of one insect per well. Plates were placed in an incubator set at 25 °C, 88% relative humidity (RH), and 0:24 light to dark hours for seven days. Larval parameters were assessed at the end of the bioassay period, including growth according to body size^46^, development according to head capsule size^88^, and mortality.

### Late-third-instar exposure bioassays

Late-third-instar bioassays were conducted in two experiments as previously described^87^, modified as appropriate for assessment of WCR core RNAi machinery knockdown. Briefly, the first was a pilot experiment containing a water negative control and *dcr-1* dsRNA. The second experiment contained ten treatments: three negative controls (water, buffer, *E. coli gus* dsRNA), and dsRNA against each of *drosha*, *dcr-2*, *pasha*, *loqs*, *r2d2*, *ago1*, and *ago2*. Each dosing solution except water treatments contained the same buffer percentage (v·v^−1^), and each dsRNA dosing solution contained unpurified dsRNAs at an estimated concentration of 300 ng·µL^−1^, yielding a final estimated diet concentration of 50 ng·µL^−1^. Four plates were prepared for each treatment, and were infested with 300–400 healthy, pre-acclimatized, eleven-day-old third instar larvae. After placement and incubation of larvae in pupation dishes, emerging adults were collected, counted, sexed, and transferred to a holding cage. After ten days the number of males and females was adjusted to a target of 16–20 pairs per cage and three cages per treatment. Each cage received oviposition dishes and eggs were collected over a period of 15 days, during which beetle mortality was also tracked. The oviposition period was divided into three time points from which eggs were collected: days 1–5, 6–10, and 11–15. At the end of each time point, total number of eggs per cage was counted and a subset of egg aliquots was utilized for seven-day hatch tests.

### Statistical analysis

Statistical analyses were conducted using SAS software (v. 9.4). Replication within the early-first-instar bioassay was at the level of individual insects, with a target of 96 per treatment per bioassay day, and treatments were compared to the buffer and *gus* controls. Replication within the late-third-instar bioassay was at the level of the pupation dish (target of 4), oviposition cage (target of 3), or egg aliquot (3 per cage per time point), and pairwise comparisons were made between all treatments. Linear mixed models or generalized linear mixed models were fit to the data as most appropriate for each observation variable. Variables which were known to inherently violate the assumptions of linear mixed models, such as survival data, were fit with generalized linear mixed models which allow for appropriate distributional assumptions. For continuous data, a linear mixed model or a linear fixed effects model was used, and graphical assessment of residuals was used to assess model fit in terms of the assumption of normally and independently distributed errors with homogeneous variance. All statistical comparisons were conducted with a two-sided test; significant differences were identified for *P*-values <α, where α = 0.05. Additional details of statistical evaluations are provided in the Supplementary Methods. All estimated metrics with associated uncertainty and significance values may be found in Supplementary File 1.

### Sample collection and RNA isolation

Insects were collected from early-first-instar bioassays for gene and miRNA expression analysis at two and seven days post-exposure. Three samples per treatment per time point were collected and weighed, at a target rate of 32 live insects per sample. Insects were collected from late-third-instar bioassays as third instar larvae, pupae, pre- and post-oviposition adult males and females, and eggs at 3, 13, 30, 45, and 56 days post-exposure, respectively. Three samples per treatment per time point were collected and weighed at a target rate of eight live insects per sample for all sample types except egg. All eggs were pooled by treatment at the end of each collection time point and weighed. Samples stored as flash-frozen homogenates were removed and thawed on the day of RNA isolation; frozen insects were homogenized on the day of RNA isolation. The RNA isolation procedure followed the manufacturer’s protocol included with the Ambion *mir*Vana miRNA Isolation Kit for separation and isolation of large (>200 nt) and small (<200 nt) RNA fractions. Directly following column elution, isolated large RNAs were DNase-treated using the RNase-free DNase kit and re-purified using the Isolate II RNA Micro Kit, both per manufacturer’s instructions. Small RNA samples were flash-frozen in liquid nitrogen and stored at −80 °C after isolation. DNase digestion occurred on a separate day using the Ambion TURBO DNA-*free* Kit and associated protocol.

### Reverse transcription and quantitative PCR

Expression levels of targeted core RNAi machinery genes were assessed using MIQE-compliant RT-qPCR assays^89^. Purified large RNAs were quantified and cDNA was prepared on the day of RNA isolation using the Invitrogen SuperScript II First-Strand Synthesis System for RT-PCR. Reaction conditions were as described in manufacturer’s instructions for a mix of both oligo-dT and random hexamer priming. Resulting cDNA was analyzed with triplex qPCR assays developed between a WCR core RNAi machinery gene and two WCR genes (*α-tubulin* and *ef1α*) previously validated as suitable RT-qPCR references^26,90^. The SensiFAST Probe Lo-ROX Kit was used to amplify DNA. In addition to bioassay samples, each sample plate contained three standard curves of synthetic DNA fragments (one per assay target) and no amplification controls (NACs). Standard curves and NACs were analyzed in triplicate; bioassay samples were analyzed in quadruplicate. Quantitative PCR was conducted using the Applied Biosystems Viia 7 Real-Time PCR System. MIQE-required information may be found in Supplementary File 2.

Expression levels of five miRNAs were evaluated using a customized IDEAL miRNA Kit with Spike-in RNA (MiRXES, Pte Ltd., Singapore). DNase-treated small RNAs were quantified and reverse-transcribed using species-specific primers. Reverse transcription reactions contained 3.1 ng small RNA and 1 µL RNA normalizer per 15 µL reaction. Resulting cDNA was stored at −80 °C, and was diluted 1:20 immediately prior to analysis. The IDEAL miRNA qPCR Master Mix was used with species-specific primers in singleplex qPCR to amplify 3.3 µL diluted cDNA in 10 µL total reaction volumes on 384-well PCR microplates. Each plate contained NACs in addition to bioassay samples—both were analyzed in triplicate. Quantitative PCR was conducted as above, with the addition of a melt curve at the end of each run. Conditions for both reverse transcription and qPCR were as instructed in manufacturer-provided protocols; sequences used for assay design are listed in Table S1.

### Expression analysis

Quantitative PCR data were generated for core RNAi transcripts using the QuantStudio Real-Time PCR Software (v. 1.1) with “Standard Curve” experimental design. Normalized expression of the target gene within each sample was determined using the geometric mean of the ratios between interpolated values of the target gene to each reference gene. Values for the median and median absolute deviation (MAD) were then calculated across all sample means within each treatment group. Further details of these calculations may be found in the Supplementary Methods. Quantitative PCR data were generated for miRNAs using the QuantStudio Real-Time PCR Software with “Comparative Ct” experimental design. Expression of miRNAs was determined relative to an artificial RNA spike, as previously described^91^. Values for the treatment average and standard deviation (SD) were calculated using all samples within each treatment group.

### Data availability

The expression datasets generated during the current study are available from the corresponding author on reasonable request. All other data generated or analyzed during this study are included in this published article and its Supplementary Information files.

## Electronic supplementary material

Supplementary Information

Supplementary File 1

Supplementary File 2

## References

[1] Ketting RF (2011). The many faces of RNAi. Developmental cell.

[2] Fabian MR, Sonenberg N, Filipowicz W (2010). Regulation of mRNA translation and stability by microRNAs. Annual review of biochemistry.

[3] Pham JW, Pellino JL, Lee YS, Carthew RW, Sontheimer EJ (2004). A Dicer-2-dependent 80s complex cleaves targeted mRNAs during RNAi in Drosophila. Cell.

[4] Ghildiyal M (2008). Endogenous siRNAs derived from transposons and mRNAs in Drosophila somatic cells. Science.

[5] Denli AM, Tops BB, Plasterk RH, Ketting RF, Hannon GJ (2004). Processing of primary microRNAs by the Microprocessor complex. Nature.

[6] Gregory RI (2004). The Microprocessor complex mediates the genesis of microRNAs. Nature.

[7] Lee YS (2004). Distinct roles for Drosophila Dicer-1 and Dicer-2 in the siRNA/miRNA silencing pathways. Cell.

[8] Forstemann K (2005). Normal microRNA maturation and germ-line stem cell maintenance requires Loquacious, a double-stranded RNA-binding domain protein. PLoS biology.

[9] Saito K, Ishizuka A, Siomi H, Siomi MC (2005). Processing of pre-microRNAs by the Dicer-1-Loquacious complex in drosophila cells. PLoS biology.

[10] Bernstein E, Caudy AA, Hammond SM, Hannon GJ (2001). Role for a bidentate ribonuclease in the initiation step of RNA interference. Nature.

[11] Liu Q (2003). R2D2, a Bridge Between the Initiation and Effector Steps of the Drosophila RNAi Pathway. Science.

[12] Schwarz DS (2003). Asymmetry in the assembly of the RNAi enzyme complex. Cell.

[13] Khvorova A, Reynolds A, Jayasena SD (2003). Functional siRNAs and miRNAs exhibit strand bias. Cell.

[14] Miyoshi K, Okada TN, Siomi H, Siomi MC (2009). Characterization of the miRNA-RISC loading complex and miRNA-RISC formed in the Drosophila miRNA pathway. Rna.

[15] Hammond SM, Boettcher S, Caudy AA, Kobayashi R, Hannon GJ (2001). Argonaute2, a link between genetic and biochemical analyses of RNAi. Science.

[16] Matranga C, Tomari Y, Shin C, Bartel DP, Zamore PD (2005). Passenger-strand cleavage facilitates assembly of siRNA into Ago2-containing RNAi enzyme complexes. Cell.

[17] Zhang J, Khan SA, Heckel DG, Bock R (2017). Next-Generation Insect-Resistant Plants: RNAi-Mediated Crop Protection. Trends Biotechnol.

[18] Guo H (2014). Plant-generated artificial small RNAs mediated aphid resistance. PLoS ONE.

[19] Agrawal A, Rajamani V, Reddy VS, Mukherjee SK, Bhatnagar RK (2015). Transgenic plants over-expressing insect-specific microRNA acquire insecticidal activity against Helicoverpa armigera: an alternative to Bt-toxin technology. Transgenic research.

[20] Baum JA (2007). Control of coleopteran insect pests through RNA interference. Nat Biotechnol.

[21] Sappington TW, Siegfried BD, Gullemaud T (2006). Coordinated Diabrotica genetics research: accelerating progress on an urgent insect pest problem. American Entomologist.

[22] U.S. -EPA. EPA Registers Innovative Tool to Control Corn Rootworm. *News Releases from Headquarters*. https://www.epa.gov/newsreleases/epa-registers-innovative-tool-control-corn-rootworm Accessed 7/30/2017. (2017).

[23] Fishilevich, E. *et al*. RNAi as a management tool for the western corn rootworm, Diabrotica virgifera virgifera. *Pest Management Science* **72**, 10.1002/ps.4324 (2016). 10.1002/ps.4324 27218412

[24] Gibson, T. L. RNA T: Human Health and Ecological Risk Assessments For SmartStax PRO. 1-50 (United States Environmental Protection Agency, Washington, D.C., 2016).

[25] Velez AM, Khajuria C, Wang H, Narva KE, Siegfried BD (2016). Knockdown of RNA Interference Pathway Genes in Western Corn Rootworms (Diabrotica virgifera virgifera Le Conte) Demonstrates a Possible Mechanism of Resistance to Lethal dsRNA. PLoS ONE.

[26] Miyata K (2014). Establishing an *in vivo* assay system to identify components involved in environmental RNA interference in the western corn rootworm. PLoS ONE.

[27] Wu K (2017). Distinct fitness costs associated with the knockdown of RNAi pathway genes in western corn rootworm adults. PLoS ONE.

[28] Obbard DJ, Jiggins FM, Halligan DL, Little TJ (2006). Natural selection drives extremely rapid evolution in antiviral RNAi genes. Current biology: CB.

[29] Casas-Mollano, J. A., Zacarias, E., Ma, X., Kim, E.-J. & Cerutti, H. In *Evolution of the Protein Synthesis Machinery and Its Regulation* (eds G. Hernandez & R. Jagus) Ch. 20, 513-529 (Springer International Publishing, 2016).

[30] Meister G (2013). Argonaute proteins: functional insights and emerging roles. Nature Reviews. Genetics.

[31] Misof B (2014). Phylogenomics resolves the timing and pattern of insect evolution. Science.

[32] Jun Tong K, Sebastián Duchêne, Simon YWHo, Lo N (2015). Comment on “Phylogenomics resolves the timing and pattern of insect evolution”. Science.

[33] Dowling, D. *et al*. Phylogenetic Origin and Diversification of RNAi Pathway Genes in Insects. *Genome Biol Evol*, 10.1093/gbe/evw281 (2017). 10.1093/gbe/evw281 PMC5521735 28062756

[34] Berry B, Deddouche S, Kirschner D, Imler JL, Antoniewski C (2009). Viral suppressors of RNA silencing hinder exogenous and endogenous small RNA pathways in Drosophila. PLoS ONE.

[35] Gammon DB, Mello CC (2015). RNA interference-mediated antiviral defense in insects. Curr Opin Insect Sci.

[36] Sabin LR, Delas MJ, Hannon GJ (2013). Dogma derailed: the many influences of RNA on the genome. Mol Cell.

[37] Gassmann AJ, Carriere Y, Tabashnik BE (2009). Fitness costs of insect resistance to Bacillus thuringiensis. Annual review of entomology.

[38] Shapiro JS (2014). Drosha as an interferon-independent antiviral factor. Proceedings of the National Academy of Sciences of the United States of America.

[39] Marques JT (2010). Loqs and R2D2 act sequentially in the siRNA pathway in Drosophila. Nat Struct Mol Biol.

[40] Okamura K, Robine N, Liu Y, Liu Q, Lai EC (2011). R2D2 organizes small regulatory RNA pathways in Drosophila. Mol Cell Biol.

[41] Forstemann K, Horwich MD, Wee L, Tomari Y, Zamore PD (2007). Drosophila microRNAs are sorted into functionally distinct argonaute complexes after production by dicer-1. Cell.

[42] Okamura K, Liu N, Lai EC (2009). Distinct mechanisms for microRNA strand selection by Drosophila Argonautes. Mol Cell.

[43] Czech B (2009). Hierarchical rules for Argonaute loading in Drosophila. Mol Cell.

[44] Yang JS (2014). Intertwined pathways for Argonaute-mediated microRNA biogenesis in Drosophila. Nucleic acids research.

[45] Davis-Vogel, C. *et al*. Identification and comparison of key RNA interference machinery from western corn rootworm, fall armyworm, and southern green stinkbug. *Submitted*. 10.1371/journal.pone.0203160 PMC6124762 30183751

[46] Hu X (2016). Discovery of midgut genes for the RNA interference control of corn rootworm. Sci Rep.

[47] Bolognesi R (2012). Characterizing the mechanism of action of double-stranded RNA activity against western corn rootworm (Diabrotica virgifera virgifera LeConte). PLoS ONE.

[48] Levine E, Oloumi-Sadeghi H (1991). Management of Diabroticite rootworms in corn. Annual review of entomology.

[49] Meinke LJ, Siegfried B, Wright AJ, Chandler LD (1998). Adult susceptibility of Nebraska western corn rootworm (Coleoptera: Chrysomelidae) populations to selected insecticides. Journal of Economic Entomology.

[50] Gassmann AJ, Petzold-Maxwell JL, Keweshan RS, Dunbar MW (2011). Field-evolved resistance to Bt maize by western corn rootworm. PLoS ONE.

[51] Hatfield SD (2005). Stem cell division is regulated by the microRNA pathway. Nature.

[52] Lucchetta EM, Carthew RW, Ismagilov RF (2009). The endo-siRNA pathway is essential for robust development of the Drosophila embryo. PLoS ONE.

[53] Park JK, Liu X, Strauss TJ, McKearin DM, Liu Q (2007). The miRNA pathway intrinsically controls self-renewal of Drosophila germline stem cells. Current biology: CB.

[54] Kataoka Y, Takeichi M, Uemura T (2001). Developmental roles and molecular characterization of a Drosophila homologue of Arabidopsis Argonaute1, the founder of a novel gene superfamily. Genes to cells: devoted to molecular & cellular mechanisms.

[55] Deshpande G, Calhoun G, Schedl P (2005). Drosophila argonaute-2 is required early in embryogenesis for the assembly of centric/centromeric heterochromatin, nuclear division, nuclear migration, and germ-cell formation. Genes & development.

[56] Meyer WJ (2006). Overlapping functions of argonaute proteins in patterning and morphogenesis of Drosophila embryos. PLoS genetics.

[57] Nakahara K (2005). Targets of microRNA regulation in the Drosophila oocyte proteome. Proceedings of the National Academy of Sciences of the United States of America.

[58] Graveley BR (2011). The developmental transcriptome of Drosophila melanogaster. Nature.

[59] Graveley, B. R. *et al*. (Department of Genetics, University of Cambridge, modMine, 2010).

[60] Kalidas S (2008). Drosophila R2D2 mediates follicle formation in somatic tissues through interactions with Dicer-1. Mechanisms of development.

[61] Wen J (2015). Adaptive regulation of testis gene expression and control of male fertility by the Drosophila hairpin RNA pathway. [Corrected]. Mol Cell.

[62] Yang H (2016). MicroRNA-dependent roles of Drosha and Pasha in the Drosophila larval ovary morphogenesis. Developmental biology.

[63] Zhu L (2015). Loqs depends on R2D2 to localize in D2 body-like granules and functions in RNAi pathways in silkworm cells. Insect Biochemistry and Molecular Biology.

[64] Zhou R (2009). Processing of Drosophila endo-siRNAs depends on a specific Loquacious isoform. Rna.

[65] Haac ME, Anderson MA, Eggleston H, Myles KM, Adelman ZN (2015). The hub protein loquacious connects the microRNA and short interfering RNA pathways in mosquitoes. Nucleic acids research.

[66] Liu X (2007). Dicer-1, but not Loquacious, is critical for assembly of miRNA-induced silencing complexes. Rna.

[67] Martin R (2009). A Drosophila pasha mutant distinguishes the canonical microRNA and mirtron pathways. Mol Cell Biol.

[68] Lim MY (2016). The Drosophila Dicer-1 Partner Loquacious Enhances miRNA Processing from Hairpins with Unstable Structures at the Dicing Site. Cell Rep.

[69] Khajuria C (2015). Parental RNA interference of genes involved in embryonic development of the western corn rootworm, Diabrotica virgifera virgifera LeConte. Insect Biochem Mol Biol.

[70] Bird LJ, Akhurst RJ (2007). Effects of host plant species on fitness costs of Bt resistance in Helicoverpa armigera (Lepidoptera: Noctuidae). Biological Control.

[71] Raymond B, Sayyed AH, Hails RS, Wright DJ (2007). Exploiting pathogens and their impact on fitness costs to manage the evolution of resistance to Bacillus turingiensis. Journal of Applied Ecology.

[72] Wang XH (2006). RNA interference directs innate immunity against viruses in adult Drosophila. Science.

[73] Wang Z (2015). Drosophila Dicer-2 has an RNA interference-independent function that modulates Toll immune signaling. Science advances.

[74] Rincón-Castro, C. D. & Ibarra, J. E. In *Biological Control of Insect* Pests (ed Ninfa M. Rosas-Garcia) Ch. 2, 29-64 (Studium Press LLC, 2011).

[75] Kolliopoulou A (2015). Transcriptome analysis of Bombyx mori larval midgut during persistent and pathogenic cytoplasmic polyhedrosis virus infection. PLoS ONE.

[76] Liu, S., Chen, Y., Sappington, T. W. & Bonning, B. C. Genome Sequence of a Novel Positive-Sense, Single-Stranded RNA Virus Isolated from Western Corn Rootworm, Diabrotica virgifera virgifera LeConte. *Genome Announc* **5**, 10.1128/genomeA.00366-17 (2017). 10.1128/genomeA.00366-17 PMC5477329 28522719

[77] Liu, S., Chen, Y., Sappington, T. W. & Bonning, B. C. Genome Sequence of Diabrotica virgifera virgiferavirus2, a Novel Small RNA Virus of the Western Corn Rootworm, Diabrotica virgifera virgifera LeConte. *Genome Announc* **5**, 10.1128/genomeA.00365-17 (2017). 10.1128/genomeA.00365-17 PMC5442382 28522718

[78] Liu, S., Chen, Y., Sappington, T. W. & Bonning, B. C. Genome Sequence of the First Coleopteran Iflavirus Isolated from Western Corn Rootworm, Diabrotica virgifera virgifera LeConte. *Genome Announc* **5**, 10.1128/genomeA.01530-16 (2017). 10.1128/genomeA.01530-16 PMC5331493 28183753

[79] Cappelle K, de Oliveira CF, Van Eynde B, Christiaens O, Smagghe G (2016). The involvement of clathrin-mediated endocytosis and two Sid-1-like transmembrane proteins in double-stranded RNA uptake in the Colorado potato beetle midgut. Insect Mol Biol.

[80] Li X, Dong X, Zou C, Zhang H (2015). Endocytic Pathway Mediates Refractoriness of Insect Bactrocera dorsalis to RNA Interference. Scientific Reports.

[81] Saleh MC (2006). The endocytic pathway mediates cell entry of dsRNA to induce RNAi silencing. Nature cell biology.

[82] Wynant N, Santos D, Van Wielendaele P, Vanden Broeck J (2014). Scavenger receptor-mediated endocytosis facilitates RNA interference in the desert locust, Schistocerca gregaria. Insect Mol Biol.

[83] Spit J (2017). Knockdown of nuclease activity in the gut enhances RNAi efficiency in the Colorado potato beetle, Leptinotarsa decemlineata, but not in the desert locust, Schistocerca gregaria. Insect Biochem Mol Biol.

[84] Garcia RA (2017). Nucleases as a barrier to gene silencing in the cotton boll weevil, Anthonomus grandis. PLoS ONE.

[85] Ho SN, Hunt HD, Horton RM, Pullen JK, Pease LR (1989). Site-directed mutagenesis by overlap extension using the polymerase chain reaction. Gene.

[86] Zhao, J. Z. *et al*. mCry3A-Selected Western Corn Rootworm (Coleoptera: Chrysomelidae) Colony Exhibits High Resistance and Has Reduced Binding of mCry3A to Midgut Tissue. *J Econ Entomol*, 10.1093/jee/tow049 (2016). 10.1093/jee/tow049 27016600

[87] Niu X (2017). Control of Western Corn Rootworm (Diabrotica virgifera virgifera) Reproduction through Plant-Mediated RNA Interference. Sci Rep.

[88] Hammack L, Ellsbury MM, Roehrdanz RL, Pikul JL (2003). Larval sampling and instar determination in field populations of northern and western corn rootworm (Coleoptera: Chrysomelidae). J Econ Entomol.

[89] Bustin SA (2009). The MIQE guidelines: minimum information for publication of quantitative real-time PCR experiments. Clinical chemistry.

[90] Rodrigues, T. B. *et al*. Validation of Reference Housekeeping Genes for Gene Expression Studies in Western Corn Rootworm (Diabrotica virgifera virgifera). *PLoS ONE* **9**, 10.1371/journal.pone.0109825 (2014). 10.1371/journal.pone.0109825 PMC4214676 25356627

[91] Livak KJ, Schmittgen TD (2001). Analysis of relative gene expression data using real-time quantitative PCR and the 2^(−ΔΔCT) method. Methods.

