# Supplementary material for: Knockdown of RNA interference pathway genes impacts the fitness of western corn rootworm
Source: Sci Rep. 2018 May 18;8:7858. doi: 10.1038/s41598-018-26129-6 (PMC5959937; doi:10.1038/s41598-018-26129-6)
Supplement: Supplementary file 1 — Supplementary Information [file 41598_2018_26129_MOESM1_ESM.pdf]

Knockdown of RNA interference pathway genes impacts the fitness of western corn rootworm

Courtney Davis-Vogel[1,2,*], Angel Ortiz[1,+], Lisa Procyk[1,+], Jonathan Robeson[1,+], Adane Kassa[1], Yiwei Wang[1,+], Emily Huang[1,+], Carl Walker[1,+], Amit Sethi[1], Mark E. Nelson[1], Dipali G. Sashital[2]

[1]DuPont Pioneer, Research and Development, 7300 NW 62nd Ave., Johnston, IA, USA
[2]Iowa State University, Roy J. Carver Department of Biochemistry, Biophysics, and Molecular Biology, 2437 Pammel Dr., Ames, IA, USA
*courtney.vogel@pioneer.com
[+]these authors contributed equally to this work

Supplementary Methods

Expression analysis calculations

Expression of core RNAi machinery genes was determined by first interpolating concentration values Q from the appropriate standard curve, then determining a ratio between the target gene (T) and each reference gene (R1 & R2) within each sample and calculating a geometric mean of the ratios using equation (1):

$$Geometric\ Mean = \frac{Q_{\mathrm{T}}}{\left(\sqrt{Q_{\mathrm{R1}} \bullet Q_{\mathrm{R2}}}\right)} \qquad (1)$$

Finally, the median and median absolute deviation of the geometric means for all samples within a treatment group were calculated (Figure 2).

Statistical analysis – early-first-instar bioassays

Larval growth

A generalized linear mixed model was fit to the data using a cumulative logit link function for the assumed multinomial distribution and the Laplace method of integral approximation. Treatment was modeled as a fixed effect, and bioassay day as a random effect. Statistical comparisons between the test and negative control treatments with Sidak's multiplicity adjustments were conducted (Figure 3).

Larval mortality

A generalized linear mixed model with a logit link function for the assumed binomial distribution and the Laplace likelihood approximation method was used to fit the data and estimate mortality rate for each treatment. Treatment was considered a fixed effect, and bioassay day a random effect. Statistical comparisons between the test and negative control treatments with Dunnett's multiplicity adjustments were conducted by testing on the odds ratio (Figure 3).

Larval development

A generalized linear mixed model with a logit link function for the assumed binomial distribution and the Laplace likelihood approximation method was used to fit the data and estimate rate of live larvae in first or second and third developmental instars per treatment. Treatment was considered a fixed effect and

bioassay day a random effect. Comparisons between the test and negative control treatments with Dunnett's multiplicity adjustments were conducted by testing on the odds ratio (Table 1).

Statistical analysis – late-third-instar bioassays

Sample Mass

A one-way linear model with fixed treatment effects was used to fit the sample mass data for each sample type, and the restricted maximum likelihood estimation (REML) method was used to estimate treatment means. Pair-wise statistical comparisons were conducted with Tukey's multiplicity adjustments (Figure S1).

Total Adult Emergence

A generalized linear mixed model with a binomial distribution, a logit link function, and the Laplace likelihood approximation method was used to fit the data and estimate rate of adult emergence for each treatment. Treatment was considered a fixed effect and replicate within treatment a random effect. Comparisons between all treatments with Tukey's multiplicity adjustments were conducted by testing on the odds ratio (Figure 4a).

Male and female adult emergence

A generalized linear mixed model with a binomial distribution, a logit link function, and the Laplace likelihood approximation method was used to fit the data and estimate either male or female proportion of adult emergence for each treatment. Treatment was considered a fixed effect. Comparisons between all treatments with Tukey's multiplicity adjustments were conducted by testing on the odds ratio (Figure S2).

Adult mortality during oviposition

A generalized linear mixed model with a binomial distribution, a logit link function, and the Laplace likelihood approximation method was used to fit the data and estimate mortality rate for each treatment. Treatment, sex, and the interaction between them were considered fixed effects. Comparisons between all treatments with Tukey's multiplicity adjustments were conducted by testing on the odds ratio (Figure 4d).

Fecundity

Four approaches were used to model egg counts relative to number of WCR adult females:

1. Eggs per starting female
   A linear mixed model was used to fit the ratio of eggs collected from replicates sampled at multiple time points to the number of females present at the beginning of the oviposition period. The REML method was used to estimate treatment means, and treatment, time point, and the interaction between them were considered fixed effects. A compound symmetry covariance structure was used for the error variance-covariance matrix to allow positive or negative error covariance among repeated samples from each replicate. Since the treatment and time point interaction was statistically significant, pair-wise statistical comparisons among all treatments within each time point were conducted with Tukey's multiplicity adjustments (Figures 5a and S3a).

2. Eggs per starting female at each time point
   A linear mixed model was used to fit the ratio of eggs collected from replicates sampled at multiple time points to the number of females alive at the start of each oviposition time point. The REML method was used to estimate treatment means, and treatment, time point and the interaction between them were considered fixed effects. A compound symmetry covariance structure was used for the error variance-covariance matrix to allow positive or negative error

covariance among repeated samples from each replicate. Since the treatment and time point interaction was statistically significant, pair-wise statistical comparisons among all treatments within each time point were conducted with Tukey's multiplicity adjustments (Figure S3b).

3. Eggs per average live female
   A linear mixed model was used to fit the ratio of eggs collected from replicates sampled at multiple time points to the average number of live females during each oviposition time period. The REML method was used to estimate treatment means, and treatment, time point and the interaction between them were considered fixed effects. A compound symmetry covariance structure was used for the error variance-covariance matrix to allow positive or negative error covariance among repeated samples from each replicate. Pair-wise statistical comparisons among all treatments across time points were conducted with Tukey's multiplicity adjustments for each time point (Figure S3c).

4. Total eggs per starting female
   A linear mixed model was used to fit the ratio of total eggs collected from replicates across all time points to the number of starting females at the beginning of the oviposition period, and the REML method was used to estimate treatment means. Treatment was considered a fixed effect. Pair-wise statistical comparisons among all treatments were conducted with Tukey's multiplicity adjustments (Figure S3d).

Hatch Rate
A generalized linear mixed model with a binomial distribution, a logit link function, and the Laplace likelihood approximation method was used to fit the hatch rate data across time and estimate hatch rate for each treatment. Treatment, time point, and the interaction between them were considered fixed effects, while replicate within treatment, replication by treatment and time point, and replication by time point by aliquot within each treatment were considered random effects. Since the treatment and time point interaction was statistically significant, pair-wise statistical comparisons among all treatments within each time point were conducted with Tukey's multiplicity adjustments (Figure 5b).

Statistical analysis – qPCR assays
To estimate efficiency of the qPCR reaction for each assay (Supplementary File 2), the linear fixed effects model (2) was applied to standard curve data across multiple plates and days for each assay and target:

$$Y_{ijk} = P_j + \beta_j X_i + \epsilon_{ijk} \tag{2}$$

where Y_{ijk} is the C_q of the kth sample of the ith concentration on the jth plate, P_j denotes the intercept of the jth plate, X_i denotes the logarithm of the ith concentration, β_j denotes the slope for the jth plate, and ε_{ijk} denotes residual where $\varepsilon_{ijk} \sim iid\ N(0, \sigma^2_{Error})$.

Slope across plates was estimated for each standard curve type. Efficiency of qPCR was determined from the slope using equation (3):

$$Efficiency = (10^{-1/slope} - 1) \cdot 100 \tag{3}$$

Confidence intervals of estimated efficiencies for each assay and target were then calculated from the confidence interval of the estimated slopes. The standard error of efficiency was calculated with the Delta method using equation (4):

$$Standard\ Error\ of\ Efficiency = \sqrt{\left(\frac{10^{-1/slope} \cdot \ln(10)}{slope^2}\right)^2} \cdot Standard\ Error\ of\ Slope \qquad (4)$$

The 95% confidence intervals of the quantification cycle at each of the different concentrations tested were also estimated across plates to show viability at different concentrations along the standard curve. The Bonferroni method was applied for multiplicity adjustment of these confidence intervals.

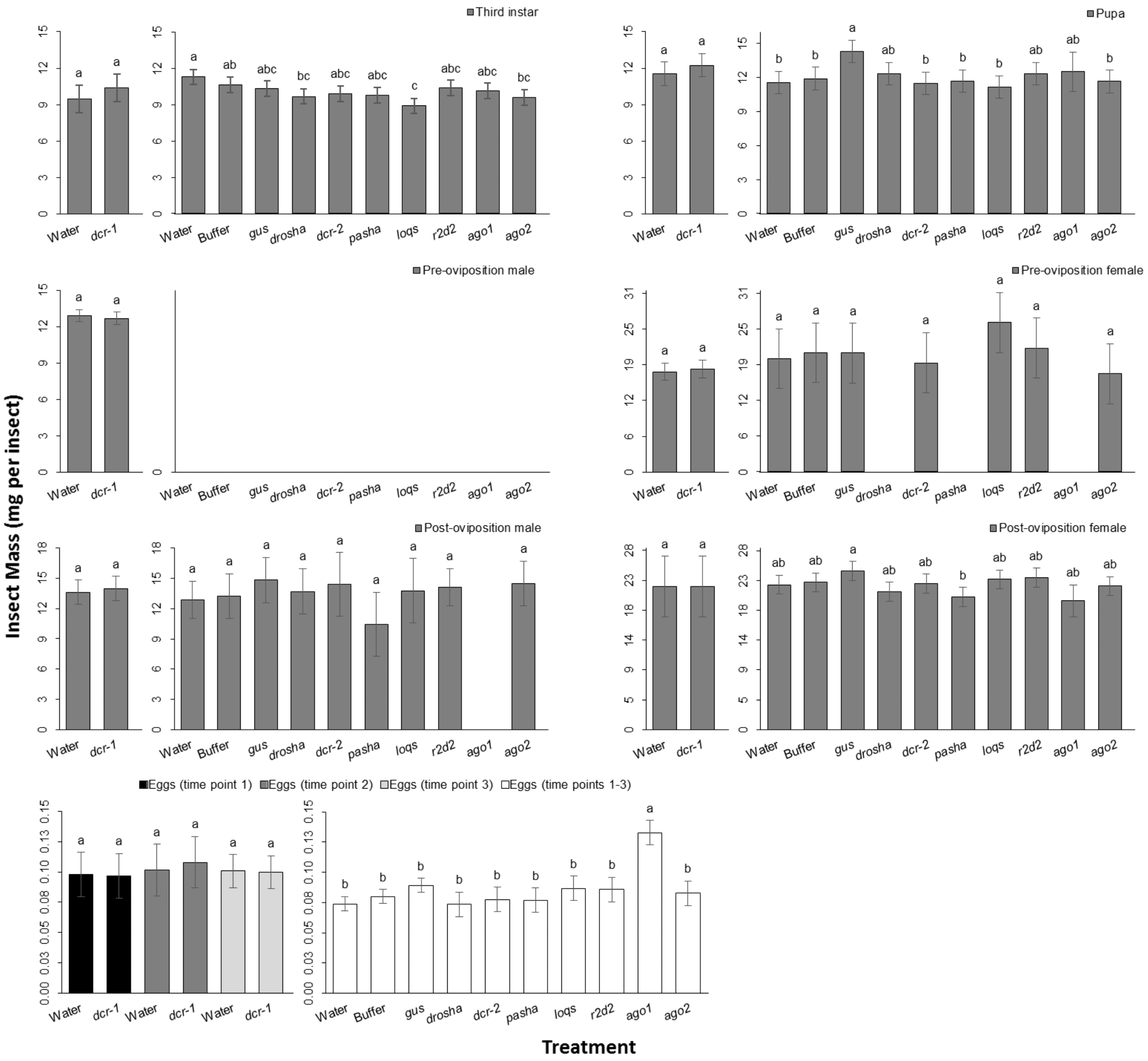

Figure S1. Mass of insects collected throughout late-third-instar bioassays. Data from both experiments of late-third-instar bioassay displayed in graphs represent means and 95% confidence intervals estimated using a one-way linear model, and statistically significant differences identified from Tukey's-adjusted *P*-values. Alphabetical letters indicating significance are shown for each treatment, and treatments followed by a common letter are not statistically different from each other at the significance level of 0.05. Missing bars indicate no insects could be spared from that treatment for sample collection. Replication within treatment consisted of a target of three samples containing eight insects each. Experiment 1 egg samples were split into three samples per time point (n=3 per time point), and for experiment 2 were kept as one sample per time point (n=3 across all time points). Results should only be compared within each experiment.

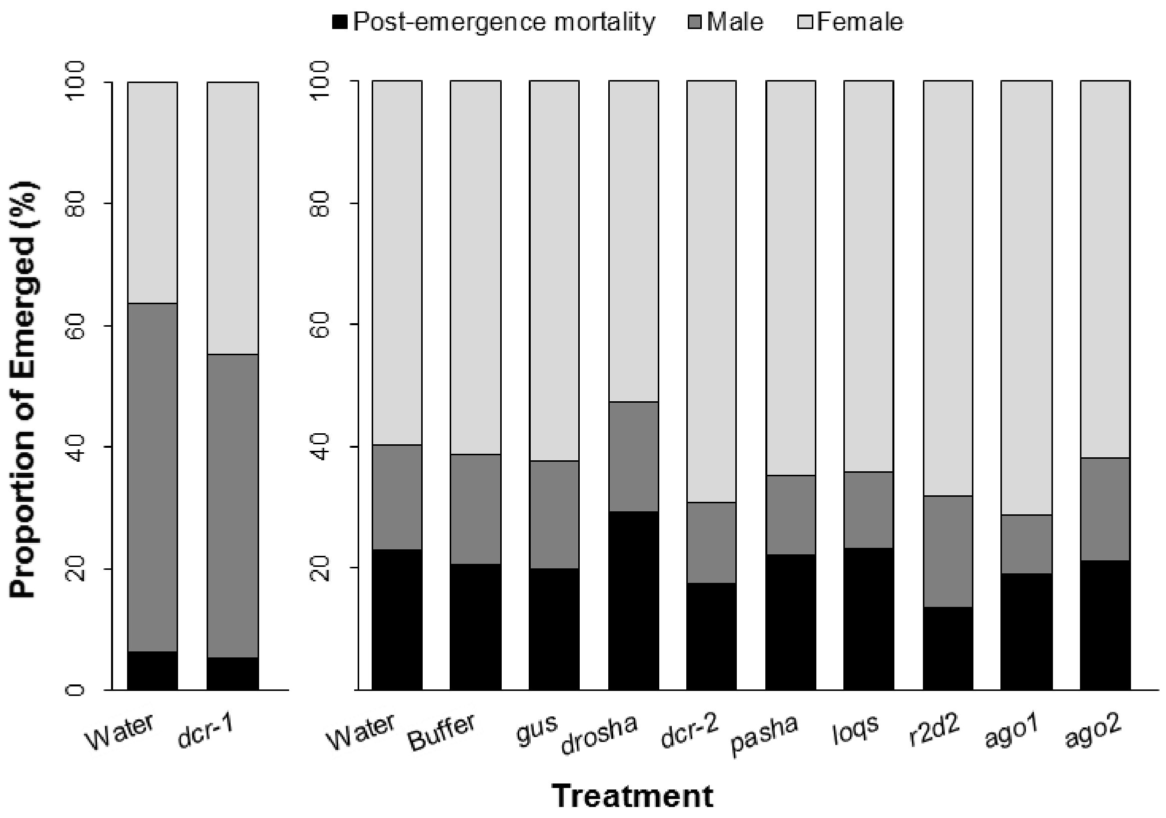

Figure S2. Proportion of each sex and post-emergence (pre-oviposition) mortality in emerged adults. Data from both experiments of late-third-instar bioassay are displayed as percent of emerged adults, and means estimated using a generalized linear mixed model are shown. No statistically significant differences were identified in post-emergence mortality or proportion of males and females of treated insects, indicated by Tukey's-adjusted *P*-values greater than 0.05. Results should only be compared within each experiment.

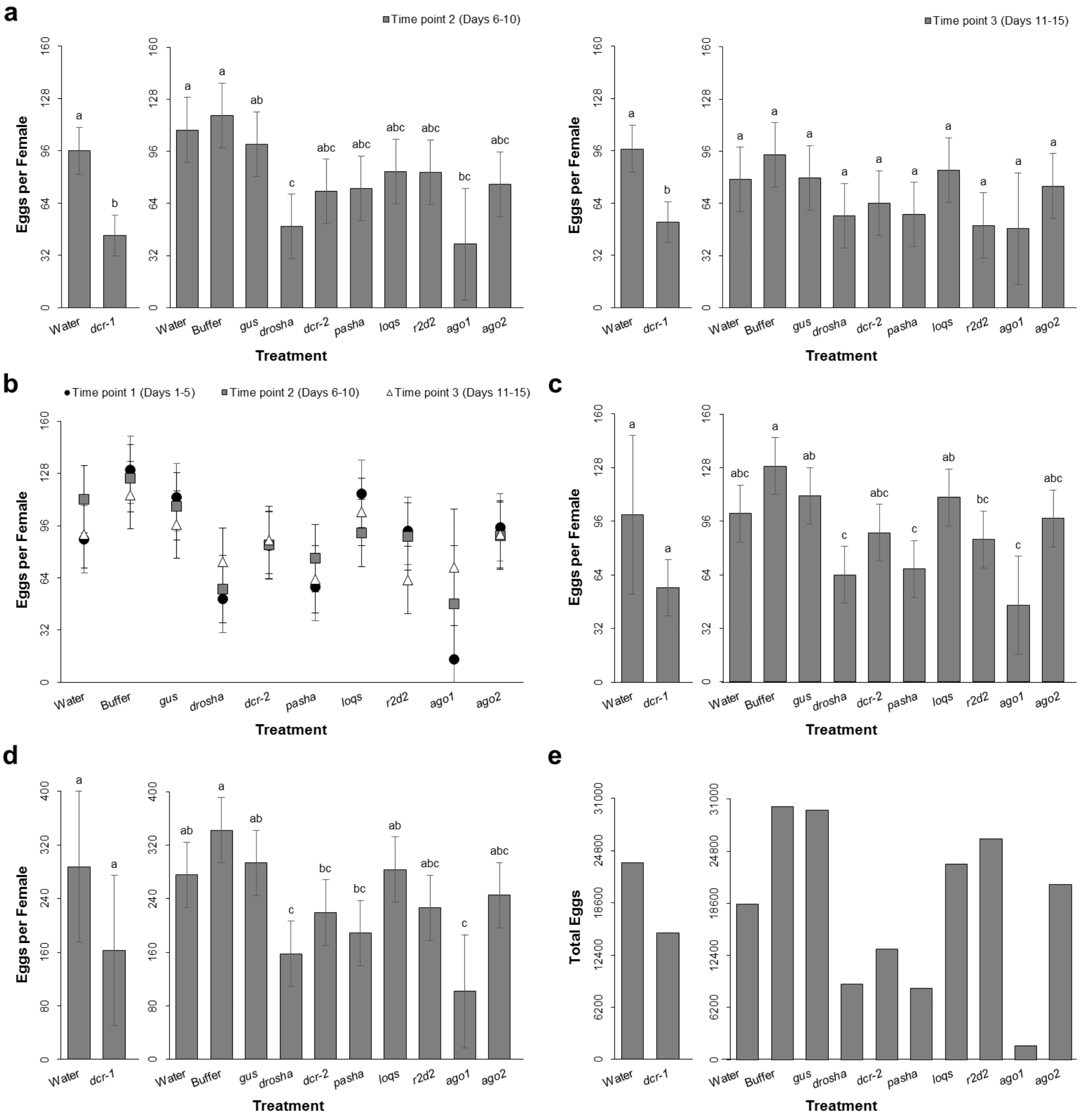

Figure S3. Egg production from females treated as third instar larvae with dsRNA against core RNAi machinery. Data from both experiments of late-third-instar bioassay displayed in graphs represent means and 95% confidence intervals estimated using a linear mixed model, and statistically significant differences identified from Tukey's-adjusted *P*-values. Alphabetical letters indicating significance are shown for each treatment, and treatments followed by a common letter are not statistically different from each other at the significance level of 0.05. Replication within treatment consisted of three oviposition cages, with the exception of the *ago1* treatment (n=1), and three time points per cage where time effects were not significant. Results should only be compared within each experiment. a) Eggs produced during indicated 5-day time point per female present at the start of oviposition for time points 2 (left) and 3 (right). b) Eggs produced during indicated time point per female alive at the start of time points 1 (black circles ●), 2 (grey squares ■), and 3 (white triangles △). Significance letters may be seen in Supplementary File 1. Data were not calculated from the first round of late-third-instar bioassay because no time effect was observed. c) Eggs produced during each 5-day time point per average number of females alive during each time point. d) Total eggs produced during the oviposition time period per female present at the start of oviposition. e) Total number of eggs produced per treatment during the entire oviposition time period (15 days).

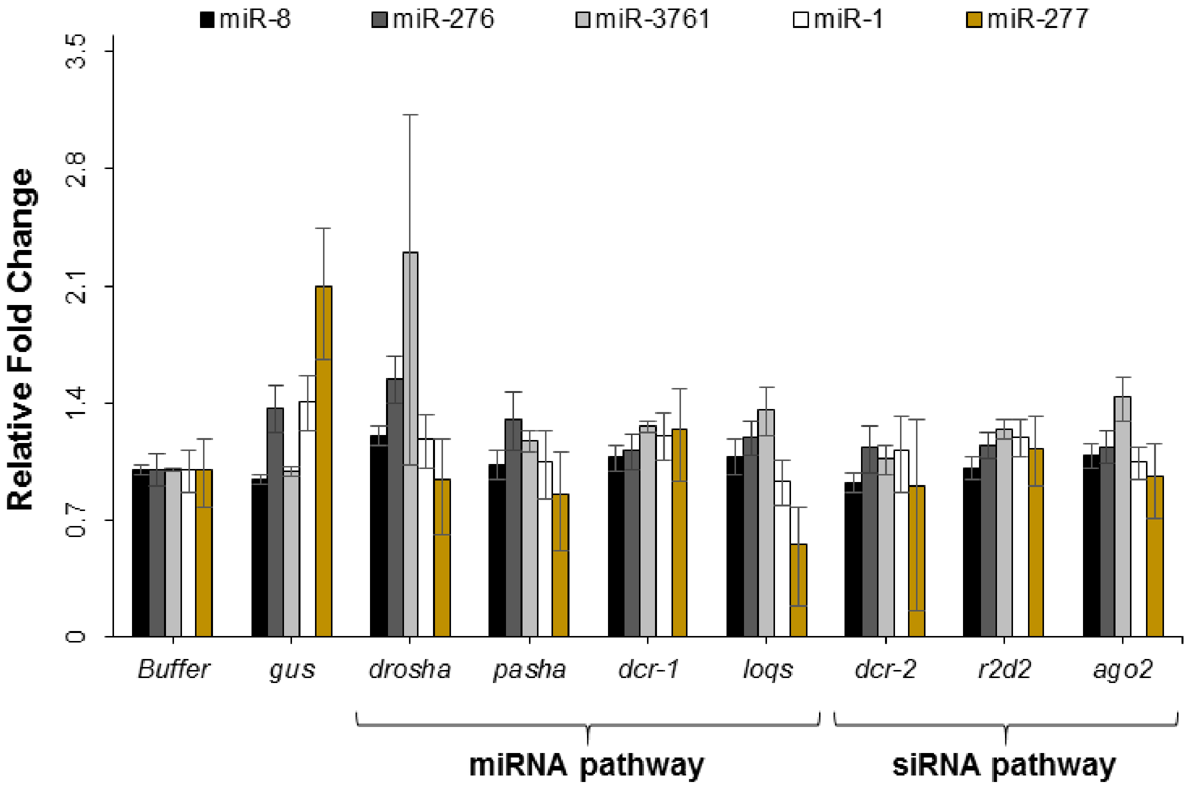

Figure S4. Effects of core RNAi machinery knockdown on miRNA expression in WCR pupae. Insects collected 13 days post-treatment in the late-third-instar bioassays were analyzed for expression of miRNAs as described in Figure 6. Insects exposed to *ago1* dsRNA were not available for analysis due to treatment effects. From left to right for each treatment, expression of miR-8 is shown in black, miR-276 in dark grey, miR-3761 in light grey, miR-1 in white, and miR-277 in gold.

Species	Sequence Name	Sequence Length (bp)	Sequence
E. coli	*gus* External IVT Forward	90	taatacgactcactatagggTGGTGATTACCGACGAAAACGGCAAGAAAA<br>AGCAGTCTTACTTCCATGATTTCTTTAACTATGCCGGAAT
	gus External IVT Reverse	90	taatacgactcactatagggATTCACCACTTGCAAAGTCCCGCTAGTGCC<br>TTGTCCAGTTGCAACCACCTGTTGATCCGCATCACGCAGT
	gus Internal IVT Forward	90	TAACTATGCCGGAATCCATCGCAGCGTAATGCTCTACACCACGC<br>CGAACACCTGGGTGGACGATATCACCGTGGTGACGCATGTCGC<br>GCA
	gus Internal IVT Reverse	90	TCCGCATCACGCAGTTCAACGCTGACATCACCATTGGCCACCAC<br>CTGCCAGTCAACAGACGCGTGGTTACAGTCTTGCGCGACATGC<br>GTC
	gus-FRAG1	277	GTGGTGATTACCGACGAAAACGGCAAGAAAAAGCAGTCTTACTT<br>CCATGATTTCTTTAACTATGCCGGAATCCATCGCAGCGTAATGC<br>TCTACACCACGCCGAACACCTGGGTGGACGATATCACCGTGGT<br>GACGCATGTCGCGCAAGACTGTAACCACGCGTCTGTTGACTGG<br>CAGGTGGTGGCCAATGGTGATGTCAGCGTTGAACTGCGTGATG<br>CGGATCAACAGGTGGTTGCAACTGGACAAGGCACTAGCGGGAC<br>TTTGCAAGTGGTGAATG
D. virgifera virgifera	*drosha*-FRAG1 External IVT Forward	90	taatacgactcactatagggTGGGCGACCACCAGTGGTACTATGACAACT<br>TAAACTATCCTCCACCTACACAAGCTCAATACAATCCCCA
	drosha-FRAG1 External IVT Reverse	90	taatacgactcactatagggTGGAGTCACCCAACAACAACACTTCGACTA<br>CAGATATTCGCACTTGCAGTCCCAATATCAAACTACAGGC
	drosha-FRAG1 Internal IVT Forward	90	TCAATACAATCCCCACATTCCACCCCAATCTAGCCACTCTTATAC<br>GCAGTGGTCTCAATCGCAACAGACATCTACGAACGTCTACAGTT<br>A
	drosha-FRAG1 Internal IVT Reverse	90	GAACGTCTACAGTTATCCTCCAGTTCCTTCGTACCCTCCTCCTC<br>CAATTCCTGCATCTTATATACCATCATCTTCTGGAGTCACCCAAC<br>A
	drosha-FRAG1	277	GTGGGCGACCACCAGTGGTACTATGACAACTTAAACTATCCTCC<br>ACCTACACAAGCTCAATACAATCCCCACATTCCACCCCAATCTA<br>GCCACTCTTATACGCAGTGGTCTCAATCGCAACAGACATCTACG<br>AACGTCTACAGTTATCCTCCAGTTCCTTCGTACCCTCCTCCTCC<br>AATTCCTGCATCTTATATACCATCATCTTCTGGAGTCACCCAACA<br>ACAACACTTCGACTACAGATATTCGCACTTGCAGTCCCAATATC<br>AAACTACAGGCG
	dcr-1-FRAG1 IVT Forward	40	taatacgactcactatagggACAAGTCCATTGTGTTCTCA
	dcr-1-FRAG1 IVT Reverse	40	taatacgactcactatagggTAGTCCAGAATTCCATTTTC
	dcr-1-FRAG1	502	GACAAGTCCATTGTGTTCTCACAACAGATAAAATAAATAATATTT<br>AGAATAATTTTAATATATTTTAAATTGTATAAATGAAATAAGGTTC<br>AAAAGAAATCTCGGATTTTCATAATGTGTTGTGTTAAATGATAAT<br>AGATATTAACCAAACTTTTTCCACTCCAAAGAGATATTTATCAAA<br>ATGGCAAGTTATCATAATGAAAATGTATACACACATACATTCACT<br>CCTAAAGAATATCAAGTTGAATTATTGGAATCTGCGAAAATCAAA<br>AACACCATAATGTGCTCCAGCACAAGTTGCGCCAAAGCTTTTAT<br>TCTTGTTAAGTTATTACAAGAATTTTCCTGGCAAATGCGAATTAA<br>GAATGGTAAAAAAGCTCTGTTTATCTTAGATCCACAGAATGTTCC<br>GATTATGACCTCGCATATAAAGTATCTAACAGATTTAAATTGTAT<br>TAGCATCATGGAATATACTCTTGATGCCAAGGAAAATGGAATTCT<br>GGACTAG
	dcr-2-FRAG1 IVT Forward	40	taatacgactcactatagggATGAGTAGCCAAGACTTGAT
	dcr-2-FRAG1 IVT Reverse	40	taatacgactcactatagggATAACTCTCGGAGGGTCAAT
	dcr-2-FRAG1	502	GATGAGTAGCCAAGACTTGATTCCTAGGAACTATCAAGTTCTTTT<br>GATGAAAATATGTCTTGAGCAAAATACTATTATTTATTTACCAACT<br>GGGTCTGGTAAAACATTTATAACTACAATGGTCCTAAAGCAAAAA<br>GGAGAAGACCTTTTAAAATCATACAGTGAGGGCGGTAAAATCTC<br>TATAATCTTAGTGAATACCGTAGCACTTGTTGATCAACATGGATC<br>TTACATTACCAATCATACAAGCTTCTCTGTTGGAAAATATACTGG<br>TGAGATGAATCTAGACTTCTGGCCAAGGACTAAATGGTTTAACG<br>AGTTTAACCAATATCAAGTATTGATAATGACGTCACAAATTTTGG<br>ATAACCTATCCAGGACTGATTATATAGATTTGAACAAAGTTAATC<br>TGTTGGTTTTTGATGAATGCCATCGAGGCGTAAACGATCATACC<br>ATGAGAAATTTAATGAAACGATTTGAACATTTGATTGACCCTCCG<br>AGAGTTATG

D. virgifera virgifera	*pasha*-FRAG1 External IVT Forward	90	taatacgactcactatagggTGTTGGAAGAGGCCTTGGAAAAACGGAAGAGAAAAGCAGCAGATGCCGGGTTAGACGACGAAGAAATTCC
	pasha-FRAG1 External IVT Reverse	90	taatacgactcactatagggATTATCTAGGCCTTATTTTCTAGGTCCAGGTTCAGCAAGAAAACACCATATACCTGTTAATGCCATACCA
	pasha-FRAG1 Internal IVT Forward	90	CGACGAAGAAATTCCATTCGAAGAAAAAAACAAGATCCTCTTAATTGAAAAAGGACAAAACCACTTCGACGTTCTTCCAGAAGGGTGGAT
	pasha-FRAG1 Internal IVT Reverse	90	TCCAGAAGGGTGGATTCAAGTAACACACAATAGTGGAATGCCTATTTTATTTACAAAAAGTTTCTAGGGTTTGTTCATTATCTAGGCCTTA
	pasha-FRAG1	277	TGTTGGAAGAGGCCTTGGAAAAACGGAAGAGAAAAGCAGCAGATGCCGGGTTAGACGACGAAGAAATTCCATTCGAAGAAAAAAACAAGATCCTCTTAATTGAAAAAGGACAAAACCACTTCGACGTTCTTCCAGAAGGGTGGATTCAAGTAACACACAATAGTGGAATGCCTATTTATTTACAAAAAGTTTCTAGGGTTTGTTCATTATCTAGGCCTTATTTTCTAGGTCCAGGTTCAGCAAGAAAACACCATATACCTGTTAATGCCATACCA
	loqs-FRAG1 External IVT Forward	90	taatacgactcactatagggTGGCCTCCATGCCGAGCAAGACTCCCGTCAGCGTCCTCCAGGAGTTGCTGAGCCGTCGCGGCATCACTCC
	loqs-FRAG1 External IVT Reverse	90	taatacgactcactatagggCTTGCTGGATCTCTTGGTCGGAAAAGTGACTCCCGAACAAGCCAATCAGACCAACGGAACGCCCGGAGCG
	loqs-FRAG1 Internal IVT Forward	90	TCGCGGCATCACTCCCAAATACGAACTGGTCCAAATCGAGGGCGCCATCCACGAGCCAATCTTCCGCTACCGCGTGTTCCTTAACAACGA
	loqs-FRAG1 Internal IVT Reverse	90	GTTCCTTAACAACGATCTGGTGGCCACCGGAACCGGAAGATCGAAGAAAGACGCCAAACATTCGGCAGCCAAGAACTTGCTGGATCTCTT
	loqs-FRAG1	277	GTGGCCTCCATGCCGAGCAAGACTCCCGTCAGCGTCCTCCAGGAGTTGCTGAGCCGTCGCGGCATCACTCCCAAATACGAACTGGTCCAAATCGAGGGCGCCATCCACGAGCCAATCTTCCGCTACCGCGTGTTCCTTAACAACGATCTGGTGGCCACCGGAACCGGAAGATCGAAGAAAGACGCCAAACATTCGGCAGCCAAGAACTTGCTGGATCTCTTGGTCGGAAAAGTGACTCCCGAACAAGCCAATCAGACCAACGGAACGCCCGGAGCGG
	r2d2-FRAG1 External IVT Forward	90	taatacgactcactatagggCCGTATTGTTTATTTTTATACAAAAGAATCTGAAAATTGTAAATCTACACAAAAATGTCAAATCATGTCA
	r2d2-FRAG1 External IVT Reverse	90	taatacgactcactatagggTCTAGCAGCTTTAATGCATTGTATGCAGCATCATGTTTACTAATCTGCTTTGAGCAACCAGTACCAGTTG
	r2d2-FRAG1 Internal IVT Forward	90	TATGAGTAATTTCATAATGTGGTGATTGAAAGCCTTTCTTTATTGCCAGCTCTTGAAGAACCATCGCCGGGGTTTTGACATGATTTGACA
	r2d2-FRAG1 Internal IVT Reverse	90	ATGAAATTACTCATAGTGTCACCGGAACTCATAACAATAGATTCGATTATAGAGTAAGAGTAGCCGGAGTGGAAGCAACTGGTACTGGTT
	r2d2-FRAG1	277	GCCGTATTGTTTATTTTTATACAAAAGAATCTGAAAATTGTAAATCTACACAAAAATGTCAAATCATGTCAAAACCCCGGCGATGGTTCTTCAAGAGCTGGCAATAAAGAAAGGCTTTCAATCACCACATTATGAAATTACTCATAGTGTCACCGGAACTCATAACAATAGATTCGATTATAGAGTAAGAGTAGCCGGAGTGGAAGCAACTGGTACTGGTTGCTCAAAGCAGATTAGTAAACATGATGCTGCATACAATGCATTAAAGCTGCTAGAG
	ago1-FRAG1 External IVT Forward	90	taatacgactcactatagggTGCCTCCAGGTTGGCCAAGAACACAAGCACACATACCTACCATTAGAAGTTTGCAACATTGTTGCGGGAC
	ago1-FRAG1 External IVT Reverse	90	taatacgactcactatagggAATTTCGGCGGGGGCAGGACCCTCCCTCTGACCTCCATCATGTTGTTGCTGATGGTCAGACCGAATTCTT
	ago1-FRAG1 Internal IVT Forward	90	TGTCCGGCGCCGATCTCGCTGTTGCTTTGATCATCGTCGAAGTCTGCATGTCCGTTAACTTCTTGATACACCTTTGTCCCGCAACAATGT
	ago1-FRAG1 Internal IVT Reverse	90	GATCGGCGCCGGACAGAGAACGCGAAATCAACAACTTGGTCCGTCGAGCCGACTTCAACAACGACGAGTACGTACAAGAATTCGGTCTGA
	ago1-FRAG1	277	GTGCCTCCAGGTTGGCCAAGAACACAAGCACACATACCTACCATTAGAAGTTTGCAACATTGTTGCGGGACAAAGGTGTATCAAGAAGTTAACGGACATGCAGACTTCGACGATGATCAAAGCAACAGCGAGATCGGCGCCGGACAGAGAACGCGAAATCAACAACTTGGTCCGTCGAGCCGACTTCAACAACGACGAGTACGTACAAGAATTCGGTCTGACCATCAGCAACAACATGATGGAGGTCAGAGGGAGGGTCCTGCCCCCGCCGAAATTG
	ago2-FRAG1 External IVT Forward	90	taatacgactcactatagggTGGGTCCACATGGAGATGTACCACAACAACCTAAATCGGCAAACGGGCAACAAAGGGGTGGACCACAACA

D. virgifera virgifera	*ago2*-FRAG1 External IVT Reverse	90	taatacgactcactatagggCAACATCATAGTGATAAGCCGTGTTTAATTTACCTAGCGCGAGGCTGAGATGATTCGTTTCTACGGGTAT
	ago2-FRAG1 Internal IVT Forward	90	TTGCAGTTCACTCATACGAGATGTCAATTCTTTAACAGAAGCTGTCTGCTGCCTATCCTGAGGCCTACGCTGTTGTTGTGGTCCACCCCT
	ago2-FRAG1 Internal IVT Reverse	90	ATGAGTGAACTGCAAGCTGGTCCACTTGTACCTATGAGGTTAAGAAACCCTGAACCTGGAAAAGCAGGTCGCAAAATACCCGTAGAAACG
	ago2-FRAG1	277	GTGGGTCCACATGGAGATGTACCACAACAACCTAAATCGGCAAACGGGCAACAAAGGGGTGGACCACAACAACAGCGTAGGCCTCAGGATAGGCAGCAGACAGCTTCTGTTAAAGAATTGACATCTCGTATGAGTGAACTGCAAGCTGGTCCACTTGTACCTATGAGGTTAAGAAACCCTGAACCTGGAAAAGCAGGTCGCAAAATACCCGTAGAAACGAATCATCTCAGCCTCGCGCTAGGTAAATTAAACACGGCTTATCACTATGATGTTGG
	miR-1	22	TGGAATGTAAAGAAGTATGGAG
	miR-8	22	CATCTTACCGGGCAGCATTAGA
	miR-276	22	TAGGAACTTCATACCGTGCTCT
	miR-277	23	TAAATGCACTATCTGGTACGACA
	miR-3761	22	TCGTTTCCCGGGCAGTGCACCA
**cDNA PCR Conditions**			
**Step No.**	**Step**	**Temp (ºC)**	**Time (min)**
1	Incubation	94	2
2	Denature	94	0.5
3	Anneal	42	0.5
4	Extend*	68	1
5	Repeat cycle from 2 x8		
6	Denature	94	0.5
7	Anneal	65	0.5
8	Extend*	68	1
9	Repeat cycle from 6 x30		
10	Final Elongation	68	10
11	Cool & Hold	4	hold
**Overlapping Primer PCR Conditions**			
**Step No.**	**Step**	**Temp (ºC)**	**Time (min)**
1	Primer incubations	94	2
2		50	5
3		94	0.5
4		50	5
5	Denature	94	0.5
6	Anneal	60	0.5
7	Extend*	72	0.75
8	Repeat cycle from 5 x35		
9	Final Elongation	75	5
10	Cool & Hold	4	hold

Table S1. Primers and amplification conditions used for production of WCR RNAs
